# Efficacy and safety of zuranolone in Japanese adults with major depressive disorder: A double‐blind, randomized, placebo‐controlled, Phase 3 clinical trial

**DOI:** 10.1111/pcn.13917

**Published:** 2025-11-18

**Authors:** Masaki Kato, Kazuyuki Nakagome, Takamichi Baba, Takuhiro Sonoyama, Hiroki Fukuju, Ryosuke Shimizu, Juan Carlos Gomez, Tomoko Motomiya, Takeshi Inoue

**Affiliations:** ^1^ Department of Neuropsychiatry Kansai Medical University Osaka Japan; ^2^ Department of Psychiatry National Center of Neurology and Psychiatry Tokyo Japan; ^3^ Drug Development and Regulatory Science Division, Shionogi & Co., Ltd. Osaka Japan; ^4^ Shionogi B.V. London UK; ^5^ Department of Psychiatry Tokyo Medical University Tokyo Japan; ^6^ Sapporo Hanazono Hospital Hokkaido Japan

**Keywords:** depressive disorder, efficacy, Japan, phase 3, zuranolone

## Abstract

**Aim:**

To evaluate the efficacy and safety of oral zuranolone for 14 days, compared with placebo, in Japanese patients with major depressive disorder (MDD).

**Methods:**

This multicenter, Phase 3 study was conducted in two parts (70 sites; Japan) including a randomized, double‐blind, placebo‐controlled, parallel‐group part presented herein. Participants aged 18–75 years with the 17‐item Hamilton Rating Scale for Depression (HAMD‐17) total score ≥ 22 were randomized (1:1) ratio to receive either zuranolone (30 mg once daily) or placebo for 14 days with follow‐up on Days 3, 8, and 15 and then weekly for 6 weeks (57 days) thereafter. The primary endpoint was change from baseline in the HAMD‐17 total score at Day 15.

**Results:**

Overall, 412 participants were randomized to receive either zuranolone 30 mg (*n* = 207) or placebo (*n* = 205). The difference in the least‐squares mean (95% confidence interval [CI]) change from baseline in the HAMD‐17 total score between groups (−1.20; 95% CI: −2.32, −0.08; *P* = 0.0365) was statistically significant on Day 15. A significant improvement in the HAMD‐17 total score was observed with zuranolone 30 mg during the early treatment period on Days 3 and 8 compared with placebo; however, no significant differences were observed between the groups at later time points, from Day 22 to Day 57. The frequency of adverse events was higher in the zuranolone group (55.1%) than in the placebo group (40.7%); however, no serious adverse events were reported.

**Conclusion:**

Zuranolone improved depressive symptoms on Day 15, compared with placebo, in Japanese patients with MDD. No new safety signals were observed.

**Clinical Trial Registration:**

jRCT2031210577.

Major depressive disorder (MDD) is a serious and debilitating illness that is also a risk factor for suicide^1^, and early treatment intervention is considered important. The estimated global prevalence of MDD in 2020 was 2470.5 cases per 100,000 population, and after adjustment for the COVID‐19 pandemic, it was 3152.9 cases per 100,000 population.^2^ In Japan, the prevalence of MDD using the Diagnostic and Statistical Manual of Mental Disorders, 4th Edition (DSM‐IV) criteria was 2.7% for 12 months and 5.7% for lifetime.^3^

While antidepressants are available for treatment of MDD, their time‐to‐response can range from weeks to months for some patients,^4^, ^5^, ^6^, ^7^, ^8^ indicating an unmet need for rapid symptom improvement.^9^

The gamma‐aminobutyric acid (GABA)‐A receptor–positive modulator zuranolone, taken orally, once daily for 14 days, offers a novel mechanism of action and has been approved by the United States (US) Food and Drug Administration (FDA) for the treatment of postpartum depression (PPD) in adults.^10^, ^11^ Zuranolone (SAGE‐217 in the US and S‐812217 in Japan) is an oral synthetic neuroactive steroid that displays a similar molecular pharmacological profile to the endogenous neuroactive steroid allopregnanolone^12^ and is currently in clinical development in Japan as an oral, once‐daily, 14‐day treatment course for adults with MDD. Zuranolone has been evaluated in several clinical trials.^13^, ^14^, ^15^, ^16^, ^17^, ^18^, ^19^ The safety and efficacy of zuranolone were assessed in patients with MDD (US [20,^17^ 30,^16^, ^17^ and 50 mg^18^ doses]; Japan^19^ [20 and 30 mg doses]). A previous Japanese double‐blind, Phase 2, placebo‐controlled study demonstrated statistically significant improvement in the symptoms of depression in patients with MDD on Day 15 after treatment with zuranolone (20 and 30 mg doses) for 2 weeks compared with placebo.^19^ Thus, the 30 mg dose was moved forward for further development.

This Phase 3 study was designed to evaluate the efficacy and safety of 14‐day treatment with zuranolone, assessed by change from baseline in the 17‐item Hamilton Rating Scale for Depression (HAMD‐17) total score, in Japanese patients with MDD compared with placebo.

## Methods

### Study design

This multicenter, Phase 3 study (jRCT2031210577) was conducted in two parts in 70 medical institutions across Japan. Part A was a randomized, double‐blind, placebo‐controlled, parallel‐group study conducted in patients with MDD. Part B was an open‐label study conducted in participants who had completed Part A, in which participants were re‐treated as required. Herein, we present the results of Part A.

**Fig. 1:**
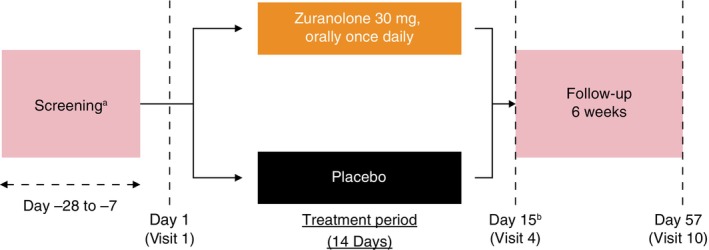
Study design. ^a^Patients were stratified at randomization according to sex, the HAMD‐17 total score at baseline (> 22 to ≤ 24 *vs*. ≥ 25), and the presence or absence of prior treatment for the current depressive episode. ^b^Primary endpoint: Change from baseline in the HAMD‐17 total score. Abbreviation: HAMD‐17, 17‐item Hamilton Rating Scale for Depression.

Part A was an 8‐week study, comprising a 2‐week treatment period after screening (1–4 weeks) and a 6‐week follow‐up period. Participants were screened for eligibility after obtaining their signed informed consent forms (ICFs). Thereafter, participants were randomized, in a 1:1 ratio, at baseline (Visit 1) to receive either zuranolone (30 mg once daily) or matching placebo, and treatment was initiated on Day 1 (Visit 1) of the 14‐day treatment period, with assessments on Days 3 (Visit 2), 8 (Visit 3), and 15 (Visit 4). Participants were stratified at randomization according to sex, the HAMD‐17 total score at baseline (≥ 22 to ≤ 24 *vs*. ≥ 25), and the presence or absence of prior treatment for the current depressive episode. After the 14‐day treatment period, study drug participants received weekly follow‐up for 6 weeks (Day 15 ± 1 [Visit 4] to Day 57 ± 2 [Visit 10]) without receiving treatment with the study drug (Fig. 1).

This study was conducted in compliance with ethical principles based on international guidelines, including the Declaration of Helsinki, Council for International Organizations of Medical Sciences (CIOMS) International Ethical Guidelines, International Council for Harmonisation (ICH) Good Clinical Practice (GCP) guidelines, and other applicable laws and regulations, and approved by the institutional review board and ethics committee of each study site (Table S1).

### Participants

Japanese outpatients aged 18–75 years at the time of signing the ICF, with a diagnosis of MDD according to the Diagnostic and Statistical Manual of Mental Disorders, 5th edition (DSM‐5) criteria, were enrolled if their current MDD episode was ongoing for at least 8 weeks but no more than 12 months prior to signing the ICF. Participants were also required to have a HAMD‐17 total score of ≥ 22 and Patient Health Questionnaire‐9 (PHQ‐9) score of ≥ 15 on Day 1.

Participants were excluded if they had evidence of serious comorbid medical conditions; had treatment‐resistant depression (no improvement in depressive symptoms with at least two different antidepressants [excluding antipsychotics] for an existing depressive episode at adequate approved doses for ≥ 4 weeks and using the Massachusetts General Hospital Antidepressant Treatment Response Questionnaire); used antidepressants within 14 days prior to Day 1 (baseline/Visit 1); were treated with device‐based therapies, such as vagal nerve stimulation, electroconvulsive therapy, and transcranial magnetic therapy, for the current depressive episode; had a comorbidity or medical history of any disease classified under DSM‐5: neurodevelopmental disorders, schizophrenia spectrum and other psychotic disorders, bipolar and related disorders, trauma‐ and stressor‐related disorders, personality disorders, obsessive‐compulsive and related disorders, anorexia nervosa, bulimia nervosa, neurocognitive disorders, and substance‐related disorders; had epilepsy (including past history), sleep apnea syndrome, interstitial pneumonia, severe bronchial asthma, alveolar hypoventilation syndrome, chronic respiratory failure, pulmonary hypertension, or other chronic respiratory diseases considered as ineligible for participation based on physician judgment; had suicidal ideation as per the Columbia‐Suicide Severity Rating Scale (C‐SSRS) within 12 months prior to or on Day 1 of randomization; and were allergic to zuranolone, allopregnanolone, or related substances.

### Intervention

Participants received 1 zuranolone 30 mg capsule or a matching placebo once daily for 14 days; the recommendation was to take the study drug within 1 h after dinner or after a light snack/meal before going to bed, if the former was not possible. The study intervention allocation was performed by using interactive response technology, and the allocations were kept blind throughout the study (until all participant data in Part B had been locked). Participant discontinuation criteria, such as liver chemistry stopping criteria, corrected QT interval (QTc) stopping criteria, study drug discontinuation due to pregnancy, or other reasons; participant discontinuation or withdrawal from the study; loss to follow‐up; prior and concomitant therapy; risk assessment of drug dependence and drug abuse, and PK evaluation are presented in Table S2.

### Outcomes

The primary endpoint was change from baseline in the HAMD‐17 total score on Day 15. Secondary endpoints included change from baseline in the HAMD‐17 total score at each time point (except Day 15); treatment response, defined as ≥ 50% reduction from baseline in the HAMD‐17 total score; and remission, defined as HAMD‐17 total score ≤ 7. Other secondary endpoints included improvement in the Clinical Global Impression–Severity of Illness (CGI‐S) score, change from baseline in the insomnia severity index (ISI) total score, incidence of treatment‐emergent adverse events (TEAEs)/treatment‐related adverse events (TRAEs) evaluated throughout the study period, and plasma zuranolone concentration.

### Statistical analysis

#### Sample size

Change from baseline in the HAMD‐17 total score on Day 15 was used to evaluate the efficacy of zuranolone (30 mg once daily) compared with placebo. In the domestic, Phase 2, dose‐finding study (1818A3731; Japanese population), the mean (standard deviation [SD]) change from baseline in the HAMD‐17 total score on Day 15 was −8.4 (6.1) with zuranolone and −6.2 (5.1) with placebo, and the difference between groups (zuranolone *vs*. placebo) estimated using the mixed‐effects model for repeated measures (MMRM) was −2.1. In this study, the number of study patients required to achieve at least 90% power in a two‐sample t‐test with a two‐sided significance level of 0.05 was calculated to be 179 per group, assuming a between‐group difference of −2.1 and conservative estimate of the common SD of 6.1 for each group. Considering the dropout rate during the treatment phase of the 1818A3731 study (the highest being 4.7%), the dropout rate for participants during the study was conservatively assumed at approximately 10% during the treatment phase, and the target number of study participants required for enrollment and randomization was set at 400 (200 participants per treatment arm).

#### Analysis set

The full analysis set comprised all participants randomly assigned to and administered at least 1 dose of the study drug and who had their HAMD‐17 scores measured at baseline and during at least one time point after treatment initiation. The full analysis set was used for the analysis of the primary and secondary efficacy endpoints.

The safety analysis set comprised all participants randomly assigned to the study drug, who took at least 1 dose of the study drug and underwent at least 1 safety assessment. The pharmacokinetic (PK) analysis set was a subset of the safety analysis set and comprised all participants who received at least 1 dose of zuranolone and who had at least 1 evaluable measurement of the plasma zuranolone concentration.

#### Efficacy analysis

In the primary analysis, change from baseline in the HAMD‐17 total score on Day 15 was compared between the zuranolone and placebo groups using an MMRM. Using all available data obtained on Days 3–57, the MMRM was applied, with change from baseline in the HAMD‐17 total scores as the response variable; the intervention group, time point, and interaction between the intervention group and time point as fixed effects; and the HAMD‐17 total score at baseline, presence or absence of prior drug for depressive episodes, and sex as covariates.

For categorical endpoints, the inverse probability‐weighted generalized estimating equation (IPW‐GEE) model^20^ was used to construct odds ratios (ORs) to compare the HAMD‐17 response and remission, and response of the CGI‐S scores. Using all available data obtained on Days 3–57, the IPW‐GEE model was applied, with the intervention group, time point, interaction between the intervention group and time point as fixed effects and the score at baseline, presence or absence of prior drug for depressive episodes, and sex as covariates. The IPW‐GEE model assumes an independent structure for the working correlation matrix. When the model was not converged due to a small number of events, data at the earliest time points (Day 3) were excluded from the IPW‐GEE model. Plasma zuranolone concentrations are presented as a scatter plot for Days 8 and 15.

All statistical tests were performed at a two‐sided significance level of 0.05, unless otherwise specified. In all secondary analyses, no multiplicity adjustment was performed. Differences between groups were nominally significant (nominal *P* < 0.05) if the 95% confidence interval [CI] of the group difference did not include the null value (0 for mean differences and 1 for odds ratios), but for the sake of convenience, if the nominal *P*‐value in the secondary analysis is less than 0.05, it is described as significant, and if it is 0.05 or more, it is described as non‐significant.

#### Safety analysis

Adverse events (AEs) were classified by System Organ Class and Preferred Terms according to the Medical Dictionary for Regulatory Activities (MedDRA) Version 24.1. Among the AEs reported in the electronic case report forms (eCRFs), TEAEs and TRAEs were included in the safety analyses. A TEAE was defined as any AE with an onset date on or after the date of the first dose of the study drug. TEAEs during the treatment period (Days 1–14) and follow‐up period (Days 15–57), including those leading to treatment discontinuation, were summarized. TEAEs assessed as related to the study drug were regarded as TRAEs. All analyses were performed using SAS version 9.4.

## Results

### Patient disposition

**Fig. 2:**
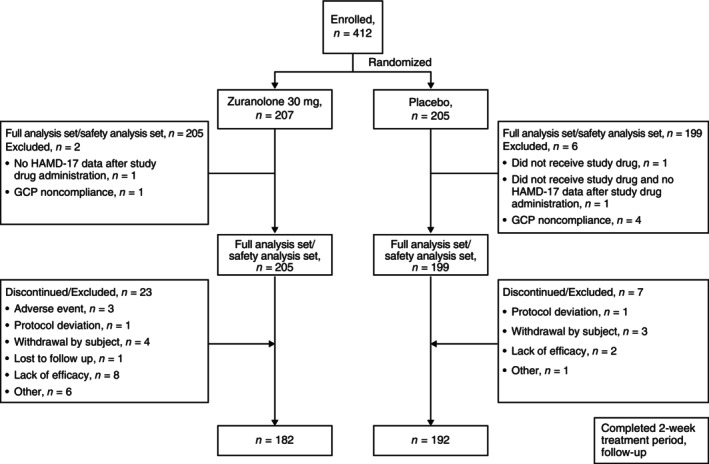
CONSORT flow chart. Abbreviations: CONSORT, Consolidated Standards of Reporting Trials; GCP, good clinical practice; HAMD‐17, 17‐item Hamilton Rating Scale for Depression.

A total of 412 participants were enrolled between March 8, 2022, and March 6, 2023, and randomized to receive zuranolone 30 mg (*n* = 207) or placebo (*n* = 205). The full analysis set/safety analysis set comprised 205 and 199 participants in the zuranolone 30 mg and placebo groups, respectively. Reasons for discontinuation/exclusion and withdrawal are presented in the CONSORT flow chart (Fig. 2). A total of 182 and 192 participants completed the 14‐day treatment period in the zuranolone 30 mg and placebo groups, respectively. During the study period (57 days), a significantly higher number of participants discontinued from the study in the zuranolone group (23/205 [11.2%]) compared with the placebo group (7/199 [3.5%]; *P* < 0.05). The most common reasons for discontinuation or exclusion were lack of efficacy (*n* = 8), followed by other reasons (*n* = 6), withdrawal by participant (*n* = 4), AEs (*n* = 3), protocol deviation (*n* = 1), and loss to follow‐up (*n* = 1) in the zuranolone 30 mg group and withdrawal by participant (*n* = 3), followed by lack of efficacy (*n =* 2), protocol deviation (*n* = 1), and other reasons (*n* = 1) in the placebo group (Fig. 2).

### Demographics and baseline characteristics

Demographics and baseline characteristics were similar between the zuranolone 30 mg and placebo groups. The mean (SD) age of participants was 39.8 (12.7) and 40.4 (12.5) years in the zuranolone 30 mg and placebo groups, respectively.

The mean (SD) baseline HAMD‐17 total score (24.1 [2.0] and 24.1 [2.1]) was ≥ 22 to ≤ 24 and ≥ 25 in 66.3% and 33.7% of participants in both the zuranolone 30 mg and placebo groups, respectively (Table 1).

**Table 1:** Demographics and baseline characteristics (full analysis set)

	Zuranolone 30 mg	Placebo
	*N* = 205 *n* (%)	*N* = 199 *n* (%)
Sex		
Male	102 (49.8)	98 (49.2)
Female	103 (50.2)	101 (50.8)
Age (years)		
≥ 18 to <25	29 (14.1)	21 (10.6)
≥ 25 to <45	103 (50.2)	98 (49.2)
≥ 45 to < 65	68 (33.2)	74 (37.2)
≥ 65	5 (2.4)	6 (3.0)
Mean (SD)	39.8 (12.7)	40.4 (12.5)
Height (cm)		
Mean (SD)	164.2 (9.0)	164.7 (9.1)
Weight (kg)		
Mean (SD)	62.0 (15.1)	62.9 (13.4)
BMI (kg/m^2^)		
Mean (SD)	22.88 (4.79)	23.07 (4.05)
Race (ethnicity)		
Asian	205 (100.0)	199 (100.0)
Not Hispanic or Latino	205 (100.0)	199 (100.0)
Employment status		
Full‐time (≥ 35 h per week)	72 (35.1)	75 (37.7)
Part‐time (< 35 h per week)	29 (14.1)	29 (14.6)
Unemployed	47 (22.9)	30 (15.1)
Retired	13 (6.3)	16 (8.0)
Other	44 (21.5)	49 (24.6)
Concurrent disease		
Yes	143 (69.8)	144 (72.4)
No	62 (30.2)	55 (27.6)
Baseline value of the HAMD‐17 total score		
Mean (SD)	24.1 (2.0)	24.1 (2.1)
≥ 22 to ≤ 24	136 (66.3)	132 (66.3)
≥ 25	69 (33.7)	67 (33.7)
Classification of MDD episodes based on DSM‐5		
Single episode	84 (41.0)	85 (42.7)
Recurrent	121 (59.0)	114 (57.3)
Episode recurrence		
First time	85 (41.5)	84 (42.2)
Second time	68 (33.2)	68 (34.2)
Third to seventh time	51 (24.9)	47 (23.6)
No less than eight times	0	0
Unknown	1 (0.5)	0
Duration of the current episode at randomization^†^		
2–4 months	76 (37.1)	75 (37.7)
4–6 months	46 (22.4)	42 (21.1)
6–8 months	33 (16.1)	38 (19.1)
8–10 months	22 (10.7)	20 (10.1)
10–12 months	23 (11.2)	22 (11.1)
> 12 months	5 (2.4)	2 (1.0)

Note: All data are in n (%) unless specified.

Abbreviations: BMI, body mass index; DSM‐5, Diagnostic and Statistical Manual of Mental Disorders, fifth edition; HAMD‐17, 17‐item Hamilton Rating Scale for Depression; MDD, major depressive disorder; SD, standard deviation.

^†^ Duration of the current episode at randomization = (Date of randomization) – (Onset date) + 1.

### Efficacy

#### Primary endpoint

**Fig. 3:**
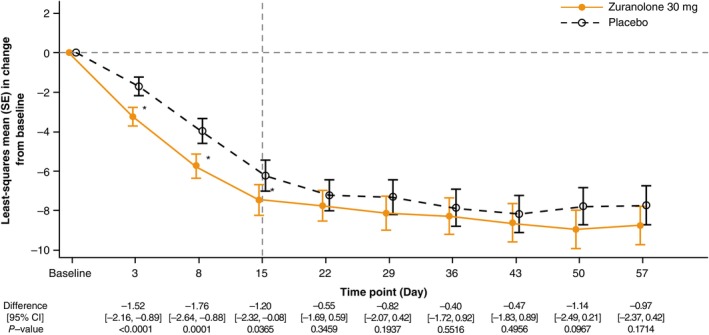
Least‐squares mean (SE) change from baseline in the HAMD‐17 total score over 57 days (full analysis set) in the zuranolone 30 mg group compared with the placebo group. MMRM with an unstructured covariance structure was applied to estimate study intervention difference between the zuranolone 30 mg and placebo groups. Fixed‐effects: treatment group, time point, and interaction effect (treatment group and time point). Covariates: baseline value of the HAMD‐17 total score, sex, and presence or absence of prior drug for depressive episodes. *P < 0.05. Abbreviations: CI, confidence interval; HAMD‐17, 17‐item Hamilton Rating Scale for Depression; MMRM, mixed‐effects model for repeated measures; SE, standard error.

The least‐squares mean (standard error [SE]) change from baseline in the HAMD‐17 total score was −7.43 (0.40) in the zuranolone 30 mg group and −6.23 (0.41) in the placebo group (Table 2 and Fig. 3), with a statistically significant difference in least‐squares mean (95% CI) between groups (−1.20; 95% CI: −2.32, −0.08; *P =* 0.0365) on Day 15. The Cohen's *d* on Day 15 was 0.21.

**Table 2:** Least‐squares mean (SE and 95% CI) change from baseline in the HAMD‐17 total score by time point over 57 days (full analysis set)

		Observed value			*Vs* placebo	
Time point (planned day)	Treatment group	*n*	Mean (SD)	Change from baseline least‐square mean (SE)	Difference of least‐squares mean [95% CI]	*P*
1	Zuranolone 30 mg	205	24.1 (2.0)	‐‐‐	‐‐‐	‐‐‐
	Placebo	199	24.1 (2.1)	‐‐‐	‐‐‐	‐‐‐
3	Zuranolone 30 mg	201	20.9 (4.0)	−3.21 (0.23)	−1.52 [−2.16, −0.89]	< 0.0001
	Placebo	199	22.4 (2.9)	−1.69 (0.23)	‐‐‐	‐‐‐
8	Zuranolone 30 mg	198	18.4 (5.0)	−5.73 (0.32)	−1.76 [−2.64, −0.88]	0.0001
	Placebo	196	20.1 (4.4)	−3.98 (0.32)	‐‐‐	‐‐‐
15	Zuranolone 30 mg	192	16.7 (5.8)	−7.43 (0.40)	−1.20 [−2.32, −0.08]	0.0365
	Placebo	194	17.8 (5.6)	−6.23 (0.41)	‐‐‐	‐‐‐
22	Zuranolone 30 mg	187	16.1 (5.9)	−7.75 (0.41)	−0.55 [−1.69, 0.59]	0.3459
	Placebo	195	16.8 (5.8)	−7.20 (0.41)	‐‐‐	‐‐‐
29	Zuranolone 30 mg	185	15.7 (6.6)	−8.13 (0.45)	−0.82 [−2.07, 0.42]	0.1937
	Placebo	192	16.7 (6.2)	−7.31 (0.45)	‐‐‐	‐‐‐
36	Zuranolone 30 mg	179	15.5 (6.8)	−8.27 (0.47)	−0.40 [−1.72, 0.92]	0.5516
	Placebo	189	16.1 (6.8)	−7.87 (0.47)	‐‐‐	‐‐‐
43	Zuranolone 30 mg	178	15.0 (7.2)	−8.63 (0.49)	−0.47 [−1.83, 0.89]	0.4956
	Placebo	188	15.9 (6.7)	−8.15 (0.49)	‐‐‐	‐‐‐
50	Zuranolone 30 mg	171	14.9 (7.1)	−8.92 (0.49)	−1.14 [−2.49, 0.21]	0.0967
	Placebo	187	16.3 (6.7)	−7.78 (0.48)	‐‐‐	‐‐‐
57	Zuranolone 30 mg	175	15.2 (7.4)	−8.71 (0.50)	−0.97 [−2.37, 0.42]	0.1714
	Placebo	184	16.3 (6.9)	−7.73 (0.50)	‐‐‐	‐‐‐

MMRM with an unstructured covariance structure was applied to estimate study intervention difference between the zuranolone 30 mg and placebo groups; Fixed effects: treatment group, time point, and interaction effect (treatment group and time point). Covariates: baseline value of the HAMD‐17 total score, sex, and presence or absence of prior drug for depressive episodes.

Abbreviations: CI, confidence interval; HAMD‐17, 17‐item Hamilton Rating Scale for Depression; MMRM, mixed‐effects model for repeated measures; SD, standard deviation; SE, standard error.

#### Secondary endpoints

A significant improvement in the HAMD‐17 total score was observed with zuranolone 30 mg during the early treatment period on Days 3 (−1.52; 95% CI: −2.16, −0.89) and 8 (−1.76; 95% CI: −2.64, −0.88) compared with the placebo group (Table 2 and Fig. 3). The change from baseline in the HAMD‐17 total scores during follow‐up (Days 22–57) did not exceed that of Day 15 in the zuranolone group (Table 2 and Fig. 3).

**Fig. 4:**
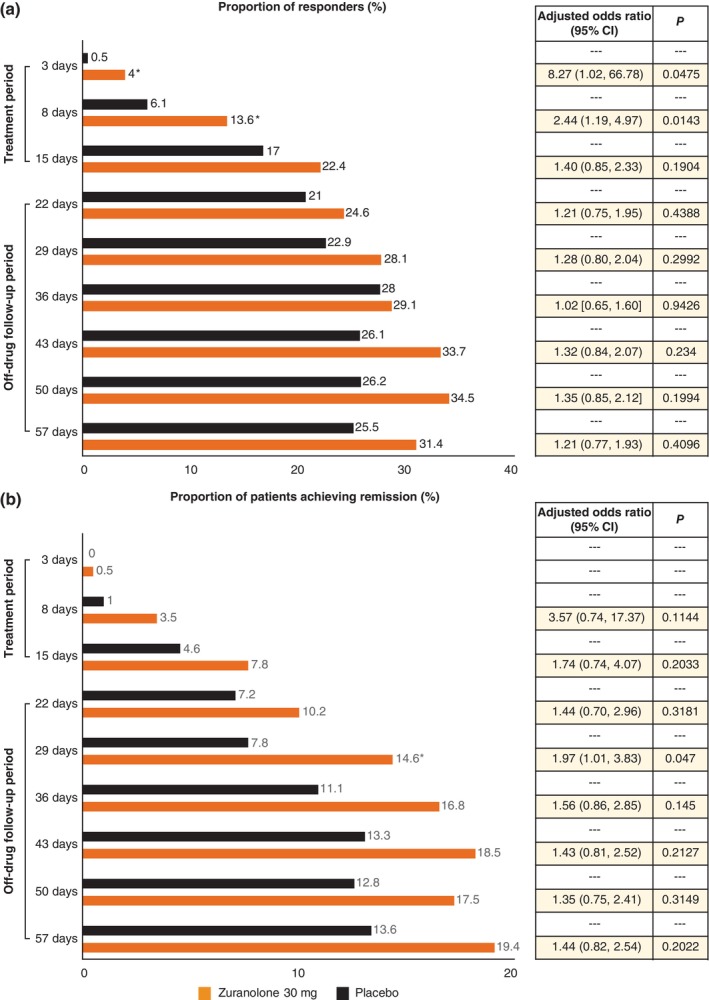
Bar graph of (A) response and (B) remission by the HAMD‐17 score over 57 days (full analysis set). *P < 0.05. IPW‐GEE analysis. IPW‐GEE with an independent structure for the working correlation matrix was applied to estimate study intervention differences between the zuranolone 30 mg and placebo groups. Fixed‐effects: treatment group, time point, and interaction effect (treatment group and time point). Covariates: baseline value of the HAMD‐17 total score, sex, and presence or absence of prior drug for depressive episodes. *P < 0.05. Response: The number of patients with nonmissing HAMD‐17 total scores at the visit and at baseline was evaluated. The number of patients with a response was defined as a ≥ 50% reduction in the baseline HAMD‐17 total score. The denominator of the percentage is the number of patients with nonmissing HAMD‐17 total score at the visit and at baseline. Remission: The number of patients with nonmissing HAMD‐17 total scores at the visit was evaluated. The number of patients with remission was defined as a HAMD‐17 score ≤ 7. The denominator of the percentage was the number of patients with nonmissing HAMD‐17 total score at the visit. In the analysis of remission, data on Day 3 were excluded from the IPW‐GEE model to avoid nonconvergence. Abbreviations: CI, confidence interval; HAMD‐17, 17‐item Hamilton Rating Scale for Depression; IPW‐GEE, inverse probability‐weighted generalized estimating equation.

In the analysis of the response rate (defined as a ≥ 50% reduction from baseline in the HAMD‐17 total score), participants receiving zuranolone 30 mg had significantly higher odds of response on Day 3 (8.27 [95% CI: 1.02, 66.78]) and Day 8 (2.44 [95% CI: 1.19, 4.97]) than those receiving placebo (Fig. 4a).

Although most time points did not show significant differences, point estimates showed that the observed remission rate was consistently higher in the zuranolone 30 mg group at each time point than in the placebo group (Fig. 4b).

The zuranolone 30 mg group reported higher remission rates than the placebo group during the treatment period on Days 8 (3.5% *vs*. 1.0%) and 15 (7.8% *vs*. 4.6%); however, the differences were not significant (*P* ≥ 0.05; Fig. 4b). After completion of the 14‐day treatment period, more participants achieved remission in the zuranolone 30 mg group than in the placebo group at all measured timepoints through Day 57; however, the differences in the OR for remission between groups were not significant (*P* ≥ 0.05; Fig. 4b).

Using the CGI‐S, as rated by the physicians, participants receiving zuranolone 30 mg had significantly higher odds of improvement in the severity of illness on Days 3 (OR: 4.55; 95% CI: 1.28, 16.16) and 8 (OR: 2.74; 95% CI: 1.47, 5.09) compared with those receiving placebo (Table S3). After completion of the 14‐day treatment period, the odds for improvement in the severity of illness using the CGI‐S remained higher in the zuranolone 30 mg group than in the placebo group at all measured timepoints through Day 57 (except on Day 36); however, the difference was not significant between groups (Table S3).

The ISI total scores were significantly lower on Days 3, 8, and 15 in the zuranolone 30 mg group than in the placebo group. The difference in the least‐squares mean (95% CI) between groups was significant during the early treatment period on Days 3 (−0.66 [−1.22, −0.10], *P =* 0.0217) and 8 (−1.32 [−2.12, −0.52], *P =* 0.013), followed by Day 15 (−1.41 [−2.36, −0.45], *P =* 0.0039; Table S4).

In Phase 3 clinical trials of zuranolone for PPD, greater decreases in depression scores were observed compared with placebo.^14^, ^15^ Therefore, to explore potential for gender‐based differences in treatment response that might suggest differential efficacy of zuranolone, a subgroup analysis was performed, which did not reveal any differences in efficacy based on gender (Fig. S1).

### Safety

Participants in this study received zuranolone for a mean (SD) of 13.5 (1.8) days (median [min, max]: 14.0 [1, 14]) and placebo for a mean (SD) of 13.9 (0.3) days (median [min, max]: 14.0 [12, 14]).

No deaths or serious TEAEs were reported during the study period. TEAEs were reported in a higher proportion of participants in the zuranolone 30 mg group (217 events in 113 participants [55.1%]) than those in the placebo group (121 events in 81 participants [40.7%]) during the study period (treatment period + follow‐up period). The most common TEAEs occurring with an incidence of ≥ 5% in the zuranolone group during the treatment period were somnolence (zuranolone 30 mg group, 13.2%; placebo group, 5.5%), dizziness (12.2% and 1.0%, respectively), and feeling abnormal (6.3% and 0.5%, respectively; Table 3). TEAEs leading to study drug discontinuation were reported in two participants (five events: two events of dizziness; one event of headache, nausea, and malaise each) in the zuranolone 30 mg group only, all of which were considered related to the study intervention, were moderate in severity, and resolved.

**Table 3:** TEAEs in ≥ 5% of participants during the treatment and follow‐up periods (safety analysis set)

	Zuranolone 30 mg (*N* = 205)		Placebo (*N* = 199)	
	Treatment period	Follow‐up period	Treatment period	Follow‐up period
System Organ Class^†^‐ Preferred Term	*n* (%)	*n* (%)	*n* (%)	*n* (%)
Participants with any TEAE	96 (46.8)	50 (24.4)	45 (22.6)	48 (24.1)
Intensity				
Mild	80 (39.0)	38 (18.5)	42 (21.1)	42 (21.1)
Moderate	16 (7.8)	12 (5.9)	3 (1.5)	6 (3.0)
Severe	0	0	0	0
TEAEs in ≥ 5% of participants				
Somnolence	27 (13.2)	0	11 (5.5)	1 (0.5)
Dizziness	25 (12.2)	1 (0.5)	2 (1.0)	1 (0.5)
Feeling abnormal	13 (6.3)	0	1 (0.5)	0

Event: Number of events.

Treatment period: up to 2 weeks; follow‐up period: 2–8 weeks.

The definition of *N* used in the follow‐up period was the same as that used in the treatment period.

AEs that occurred after discontinuation in the treatment period were counted as events in the follow‐up period.

Abbreviations: AE, adverse event; COVID‐19, coronavirus disease 2019; MedDRA, Medical Dictionary for Regulatory Activities; TEAE, treatment‐emergent adverse event.

^†^ System Organ Class and Preferred Term as per MedDRA version 24.1.

When categorized into the 14‐day treatment period (Day 1‐Day 15 ± 1) and follow‐up period (Day 15 ± 1‐Day 57 ± 2), TEAEs were observed in 46.8% (144 events in 96 patients) and 24.4% (73 events in 50 patients) of patients in the zuranolone 30 mg group and 22.6% (57 events in 45 patients) and 24.1% (64 events in 48 patients) of patients in the placebo group, respectively. A complete list of TEAEs is provided in Table S5.

TRAEs were reported in a higher proportion of patients in the zuranolone 30 mg group (108 events in 70 patients [34.1%]) than those in the placebo group (34 events in 24 patients [12.1%]).

When categorized into the 14‐day treatment period (Day 1‐Day 15 ± 1) and follow‐up period (Day 15 ± 1‐Day 57 ± 2), TRAEs were observed in 34.1% (105 events in 70 patients) and 1.5% (three events in three patients) of patients in the zuranolone 30 mg group and 11.1% (30 events in 22 patients) and 1.5% (four events in three patients) of patients in the placebo group, respectively. A complete list of TRAEs is presented in Table S6.

Based on the assessment of the Drug Dependence Assessment Committee, none of the participants met the drug dependence criteria. The Committee reviewed these data along with participants' clinical conditions; Drug Effect Questionnaire‐5 (DEQ‐5) data; efficacy data, including HAMD‐17 and PHQ‐9 total scores; and other safety data, which determined that no cases were considered drug dependence.

### Pharmacokinetics

**Fig. 5:**
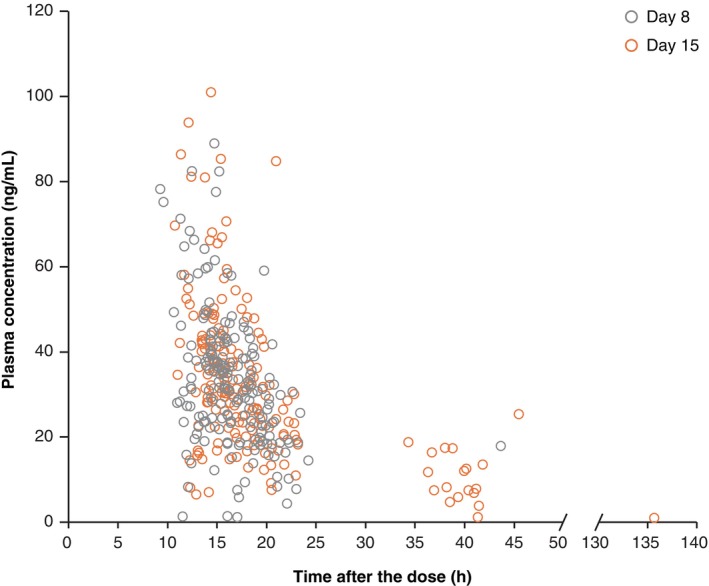
Scatter plot of plasma concentration (ng/mL) of zuranolone 30 mg on Day 8 (Visit 3) and Day 15 (Visit 4).

Zuranolone was absorbed orally, and its plasma concentrations in the zuranolone 30 mg group were comparable (steady state) between Days 8 and 15 (Fig. 5). Compared with the Phase 2 study,^19^ no change in exposure was observed in patients with diverse background factors in this study.

### Sensitivity analysis

Sensitivity analysis using tipping point analysis confirmed that our results for the primary endpoint are robust to alternative plausible missing data assumptions, as the significance was lost from the copy placebo condition^21^ (Table S7).

## Discussion

This multicenter, randomized, double‐blind, placebo‐controlled, parallel‐group part of the Phase 3 study was conducted in Japanese patients with MDD to evaluate the efficacy and safety of zuranolone 30 mg once daily for 14 days *versus* placebo using the change from baseline in the HAMD‐17 total score. The discontinuation rate was significantly higher (*P* < 0.05) in the zuranolone group (11.2% [(23/205]) compared with the placebo group (3.5% ([7/199]) by Day 57, yet similar to that of other antidepressants.^22^, ^23^ In this study, the superiority of zuranolone 30 mg over placebo was established as the difference in the least‐squares mean (95% CI and SE) of the change from baseline in the HAMD‐17 total score between the zuranolone 30 mg and placebo groups, which was statistically significant on Day 15. A significant improvement in the HAMD‐17 total score was observed in the zuranolone 30 mg group during the early treatment period on Days 3 and 8 compared with the placebo group. Sensitivity analysis using tipping point analysis confirmed the robustness of the primary endpoint results against alternative plausible missing data assumptions, making this the second study to demonstrate the efficacy of zuranolone on Day 15 in Japanese patients.^19^ The copy placebo, or copy control, approach is generally used to assess the robustness of results using an estimand framework to understand the potential systematic effect on the estimate of treatment effect of events such as dropout and switching.^21^

A placebo response was observed, as seen in many antidepressant studies. The decrease in the HAMD‐17 total score was significantly greater in the zuranolone 30 mg group than in the placebo group on Days 3, 8, and 15; however, there was no significant difference at later time points, from Day 22 until Day 57, similar to previous studies.^16^, ^17^ These results were also reflected in the response and remission rates. The response rate in the zuranolone 30 mg group was significantly higher than that in the placebo group on Days 3 and 8; however, no significant difference was observed on Day 15 or at subsequent time points. These findings suggest that zuranolone ameliorated depressive symptoms as early as Day 3 during the early phase of treatment, although the remission rate did not improve significantly compared with placebo. Time for antidepressant action can range from weeks to months in some patients^24^ and can be a problem. In contrast, zuranolone ameliorated depressive symptoms during the initial 15 days, suggesting a therapeutic benefit.

Comparisons between the treatment effect size for depression with zuranolone and individual antidepressants is challenging due to lack of meta‐analysis data of Day 3 and Weeks 1 and 2. However, Japanese MDD studies (escitalopram,^25^ paroxetine,^26^ vortioxetine,^27^ mirtazapine,^28^ duloxetine,^29^ and venlafaxine^30^) suggest that zuranolone may be beneficial in improving symptoms in the early phase of treatment. Phase 3 clinical trials of zuranolone in PPD showed greater reductions in depression scores compared with placebo and, consequently, a greater effect size.^14^, ^15^ A possible reason is that the concept of MDD includes peripartum‐onset PPD according to the definition of DSM‐5, and PPD represents a more homogeneous subgroup of MDD, comprising all women with a common trigger—pregnancy—for depressive symptoms. In this study, subgroup analysis did not reveal any differences in efficacy in MDD based on gender.

No deaths or serious AEs were reported during the study period. During the 14‐day treatment period, the most common TEAEs occurring with an incidence of ≥ 5% in the zuranolone group were somnolence (zuranolone 30 mg group, 13.2%; placebo group, 5.5%), dizziness (12.2%; 0.5%), and feeling abnormal (6.3%; 0.5%). The most common AEs reported with the 30‐mg dose in the Phase 2 study conducted in Japanese patients with MDD were somnolence (20.7%), dizziness (9.8%), nasopharyngitis (8.5%), and headache (6.1%).^19^ On the basis of the pharmacologic properties of zuranolone as a positive allosteric modulator of GABA_A_ receptors, somnolence and dizziness may be expected adverse events, consistent with previous studies.^16^, ^17^, ^18^, ^19^ In addition, the frequency of nausea and vomiting was low with zuranolone, which may be a difference compared to reports with other antidepressants.^31^ The incidence of study drug discontinuation due to TEAEs was 1% in the zuranolone group.

Studies have reported concerns around withdrawal symptoms after antidepressant discontinuation, which are akin to those observed with the discontinuation of benzodiazepines.^32^ In this study, zuranolone was not observed to overtly cause post‐discontinuation symptoms. There are no previous reports of dependence in domestic Japanese studies. Nonetheless, according to the US FDA and the Department of Justice, Drug Enforcement Administration, zuranolone has the potential to produce physical dependence similar to that of the neuroactive steroid brexanolone and benzodiazepines.^33^ Benzodiazepine medications, which interact with GABA_A_ receptors, are known to cause withdrawal symptoms such as insomnia, anxiety, restlessness, headache, nausea, and vomiting, appearing 4–7 days after stopping long half‐life benzodiazepines.^13^, ^34^ The half‐life of zuranolone is similar to that of intermediate half‐life benzodiazepines, and it is likely that withdrawal symptoms would appear within the first week after zuranolone administration is stopped. During the follow‐up period, several AEs were reported slightly more frequently in the zuranolone 30 mg group compared with the placebo group. Some of these events —headache (1.5% [3/205] *vs*. 0.5% [1/199]), diarrhea (2.4% [5/205] *vs*. 0.5% [1/199]), myalgia (2.0% [4/205] *vs*. 0.5% [1/199]), and pyrexia (3.4% [7/205] *vs*. 1.5% [3/199])—resembled withdrawal symptoms previously associated with benzodiazepines or with zuranolone discontinuation in healthy volunteers, although the difference was limited to 1–2%. When narrowed down to events occurring within 2 weeks of treatment cessation, the rates were 2.0% (4/199) *vs*. 0.5% (1/199) for myalgia and 1.5% (3/199) *vs*. 0% (0/199) for pyrexia. The possibility that they represent withdrawal effects cannot be definitively excluded. According to the package insert for ZURZUVAE in the US, adverse reactions reported upon zuranolone discontinuation in healthy participants who received 50 mg of zuranolone for 5–7 days included insomnia, palpitations, decreased appetite, nightmare, nausea, hyperhidrosis, and paranoia, suggesting potential physical dependence.^11^ Withdrawal symptoms might not have been observed in this study due to 1) lower dosing than 50–100 mg, 2) absence of specific assessment scale, or 3) masking by moderate to severe depression in participants. Further research is needed to understand the potential for withdrawal symptoms in Japanese patients. In addition, GABA modulators such as benzodiazepines are associated with misuse and abuse. A previous study showed that supratherapeutic doses of zuranolone have mild abuse liability properties, similar to alprazolam, a Schedule IV benzodiazepine.^33^

Findings from nonclinical studies in mice comparing the neuroactive steroid allopregnanolone and the benzodiazepine diazepam in the social interaction test (SIT) in the social defeat stress (SDS) model support the notion that the unique mechanism of neuroactive steroids may contribute to their potential for rapid antidepressant efficacy in depression. Allopregnanolone showed antidepressant‐like effects in the SIT in SDS model mice by decreasing the intervals of repetitive social interaction (inter‐event intervals), resulting in an increase in the total social interaction time.^35^ To date, novel antidepressant candidates such as zuranolone mostly exhibit novel mechanisms of action by acting on both synaptic and extrasynaptic GABA_A_ receptors, which may explain their unique specific contribution to depression treatment.^10^, ^35^, ^36^ Zuranolone has been shown to ameliorate symptoms as early as Day 3 of treatment in patients with MDD^17^, ^18^, ^19^ and in those with PPD.^14^, ^15^ The mechanism of action and preclinical data suggest that the effects of zuranolone may occur due to the increased theta oscillations in the basolateral amygdala and medial prefrontal cortex through potentiation of both synaptic and extrasynaptic (δ‐subunit‐containing) GABA_A_ receptors,^37^ which is thought to have an effect on brain network connectivity and function in areas associated with depression.^38^, ^39^ Symptomatic improvement at an early stage of treatment is a highly desirable characteristic. Although the positioning of zuranolone in clinical practice is yet to be determined, it may help clinicians and patients in potentially achieving the amelioration of symptoms in the early phase of treatment.

The current sample size was set to obtain a sufficient effect size when compared with a placebo at 15 days, but it may be too small to compare longer‐term differences or adverse events. Therefore, in the future, a larger sample size or pooled analysis will be necessary to evaluate the long‐term efficacy. The results of repeated treatments will be evaluated in the future; hence, conclusions regarding long‐term or repeated efficacy cannot be made. Future studies conducted in real‐world populations may provide additional data on the effects of zuranolone. In addition, participants with defined suicidal ideation were excluded from the study. Due to the use of stringent inclusion and exclusion criteria, the results may not be generalizable to all patients with MDD.

## Conclusions

This Phase 3 study demonstrated that a 14‐day treatment with zuranolone 30 mg improved the symptoms of depression on Day 15 in Japanese patients with moderate to severe MDD as compared with placebo. Treatment effect size for depression using differences in the HAMD‐17 total score favored zuranolone from Day 3 to Day 15. After completion of the 14‐day treatment, the change from baseline in the HAMD‐17 total scores on Days 22–57 did not exceed that of Day 15 in the zuranolone group. This study suggests that zuranolone may be beneficial in improving symptoms in the early phase of treatment of depression, showing its potential as an alternative symptomatic therapy to currently available drugs. Overall, all TEAEs were nonserious, and most participants experienced mild‐to‐moderate TEAEs. Treatment discontinuation due to TEAEs was low, and no new safety signals were observed.

## Author contributions

M.K., K.N., T.I., T.S., J.C.G., and T.M.: conception and design, interpretation of results, and review of the manuscript. H.F.: conception and design, acquisition and analysis of data, and review of the manuscript. T.B. and R.S.: conception and design, analysis of data, and review of the manuscript.

## Disclosure statement

T.S., T.B., and R.S. are full‐time employees and own stocks *via* employee stock ownership society of Shionogi & Co., Ltd. T.M. and H.F. are full‐time employees of Shionogi & Co., Ltd. J.C.G. is a full‐time employee of Shionogi B.V. T.I. has received personal fees from Mochida Pharmaceutical, Takeda Pharmaceutical Co., Ltd., Janssen Pharmaceuticals, Novartis Pharma, MSD, Yoshitomiyakuhin, Nipro, Kyowa Pharmaceutical Industry, Viatris, Lundbeck Japan K.K., Boehringer Ingelheim, Ono Pharmaceutical, and Meiji Seika Pharma Co., Ltd.; grants from Daiichi Sankyo and Tsumura; grants and personal fees from Shionogi & Co., Ltd., Otsuka Pharmaceutical Co., Ltd., Sumitomo Pharma Co., Ltd., Mitsubishi Tanabe Pharma, and Eisai; and is a member of the advisory boards of Luye, Shionogi, GlaxoSmithKline, Viatris, and Otsuka Pharmaceutical. M.K. has received grants from AMED, the Japanese Ministry of Health, Labour and Welfare, the Japan Society for the Promotion of Science, SENSHIN Medical Research Foundation, the Japan Research Foundation for Clinical Pharmacology, and the Japanese Society of Clinical Neuropsychopharmacology; consulting fees from Shionogi & Co., Ltd., Sumitomo Pharma Co., Ltd., Otsuka Pharmaceutical Co., Ltd., Lundbeck Japan K.K., and Takeda Pharmaceutical Co., Ltd.; speaker honoraria from Sumitomo Pharma Co., Ltd., Otsuka Pharmaceutical Co., Ltd., Lundbeck Japan K.K., Takeda Pharmaceutical Co., Ltd., Meiji Seika Pharma Co., Ltd., Shionogi & Co., Ltd., Mitsubishi Tanabe Pharma Corporation, Viatris Inc., Eisai Co., Ltd., and Kyowa Pharmaceutical Industry Co. Ltd.; and is in the general management committee for Depression Treatment Guidelines, Japan Society of Mood Disorder and the Vice Chairman of the Guideline Development Committee Japan Society of Mood Disorders in the past 36 months. K.N. has received grants paid to his institution from Shionogi & Co., Ltd., Sumitomo Pharma Co., Ltd., Otsuka Pharmaceutical Co., Ltd., Janssen Pharmaceutical K.K., Nippon Boehringer Ingelheim Co., Ltd., and AbbVie GK; honoraria from Sumitomo Pharma Co., Ltd. (Speaker, Chair, Advisor), Otsuka Pharmaceutical Co., Ltd. (Speaker, Chair, Advisor), Meiji Seika Pharma Co., Ltd. (Speaker), Janssen Pharmaceutical K.K. (Chair, Speaker‐Panelist), Mitsubishi Tanabe Pharma Corp. (Chair, Advisor), Viatris Pharmaceuticals Japan G.K. (Chair, Consulting), Nippon Boehringer Ingelheim Co., Ltd. (Chair, Advisor, Supervisor), Boehringer Ingelheim International GmbH (Speaker‐Panelist), Kyowa Kirin Co., Ltd. (Speaker), Shionogi & Co., Ltd. (Chair), and Yoshitomiyakuhin Corp. (Chair, Supervisor); and support for transportation to attend meetings from Sumitomo Pharma Co., Ltd., Otsuka Pharmaceutical Co., Ltd., Meiji Seika Pharma Co., Ltd., Janssen Pharmaceutical K.K., Mitsubishi Tanabe Pharma Corp., Nippon Boehringer Ingelheim Co., Ltd., Boehringer Ingelheim International GmbH, Shionogi & Co., Ltd., Yoshitomiyakuhin Corp., and AbbVie GK.

## Supporting information


**Figure S1.** Forest plot of subgroup analysis of change from baseline in HAMD‐17 total score at Day 15 in Part A.
**Table S1.** List of institutional review boards.
**Table S2.** Discontinuation of study drug and patient discontinuation/withdrawal criteria.
**Table S3.** Analysis of CGI‐S by timepoint (full analysis set).
**Table S4.** Changes from baseline in the ISI total score by timepoint (full analysis set).
**Table S5.** TEAEs during the treatment and follow‐up periods (safety analysis set).
**Table S6.** Overall summary of the incidence of TRAEs categorized by the treatment and follow‐up period (safety analysis set).
**Table S7.** Tipping point analysis for missing data of HAMD‐17 total score at Day 15.

## Data Availability

Shionogi & Co., Ltd. is committed to disclosing the synopses and results of its clinical trials and sharing clinical trial data (raw dataset or study data tabulation model dataset) with researchers upon reasonable request. If the research proposal is reviewed by an independent review panel and approved, the anonymized data and redacted documents will be provided in a secure research environment. For further details, please refer to the websites of Shionogi & Co., Ltd. (https://www.shionogi.com/global/en/company/policies/shionogi-group-clinical-trial-data-transparency-policy.html) and Vivli (https://vivli.org/).
