# Supplementary material for: Efficacy and safety of zuranolone in Japanese adults with major depressive disorder: A double‐blind, randomized, placebo‐controlled, Phase 3 clinical trial
Source: Psychiatry Clin Neurosci. 2025 Nov 18;80(1):76–86. doi: 10.1111/pcn.13917 (PMC12757765; doi:10.1111/pcn.13917)
Supplement: Supplementary file 1 — Figure S1. Forest plot of subgroup analysis of change from baseline in HAMD‐17 total score at Day 15 in Part A. Table S1. List of institutional review boards. Table S2. Discontinuation of study drug and patient discontinuation/withdrawal criteria. Table S3. Analysis of CGI‐S by timepoint (full analysis set). Table S4. Changes from baseline in the ISI total score by timepoint (full analysis set). Table S5. TEAEs during the treatment and follow‐up periods (safety analysis set). Table S6. Overall summary of the incidence of TRAEs categorized by the treatment and follow‐up period (safety analysis set). Table S7. Tipping point analysis for missing data of HAMD‐17 total score at Day 15. [file PCN-80-76-s001.docx]

**Supplementary data**

**Figure S1:** Forest plot of subgroup analysis of change from baseline in HAMD-17 total score at Day 15 in Part A


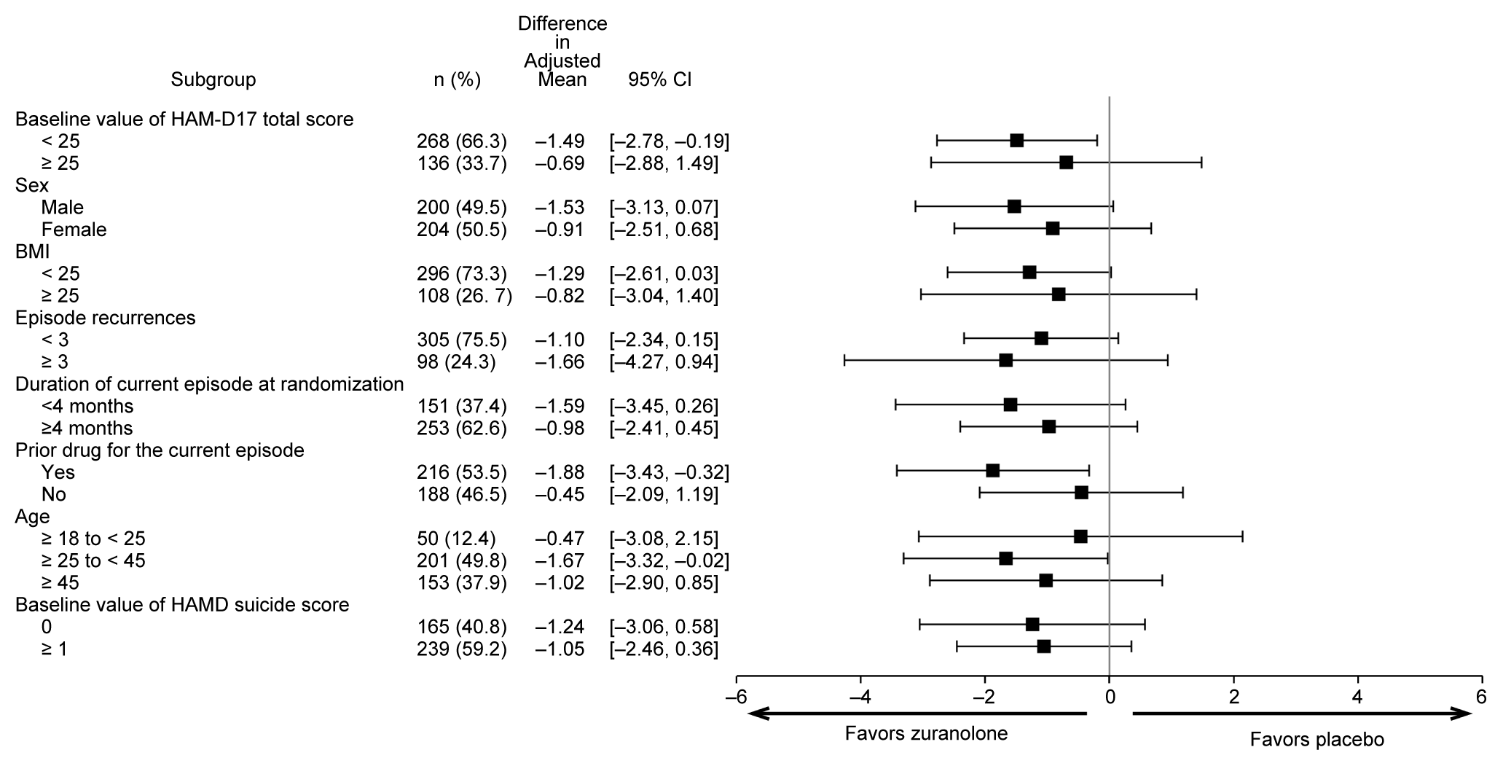

CI, confidence interval; HAMD-17, 17-item Hamilton Rating Scale for Depression

**Table S1:** List of institutional review boards

| Study center name | Name of institutional review board/institutional ethics committee |
| --- | --- |
| Tokyo Metropolitan Matsuzawa Hospital | Tokyo Metropolitan Matsuzawa Hospital Institutional Review Board |
| Maynds Tower Mental Clinic | Suzuki Internal &Circulatory Medical Clinic Institutional Review Board |
| Uguisudani Mental Clinic |  |
| Minami-Aoyama Antique Street Clinic |  |
| Kitano Mental Clinic |  |
| Kaedenomori Mental Clinic |  |
| Sangenjaya Nakamura Mental Clinic |  |
| Sangenjaya Neurology-Psychosomatic Clinic |  |
| Senzoku Psychosomatic Medicine Clinic |  |
| Tamaki Clinic |  |
| Yui Mental Clinic | Nihonbashi Sakura Clinic Institutional Review Board |
| Medical Corporation Ryokuwakai STRESS CARE HIBIYA CLINIC |  |
| Nishi-shinjuku Concieria Clinic | Mizuo Clinic Institutional Review Board |
| Kishiro Mental Clinic |  |
| Ichigaya Himorogi Clinic | Ichigaya Himorogi Clinic Institutional Review Board |
| Hanamaru Mental Clinic | Takahashi Psychiatric Clinic Institutional Review Board |
| Jin Mental Clinic |  |
| Inoue Clinic |  |
| Shibamoto Clinic |  |
| Takahashi Psychiatric Clinic |  |
| Watanabe Clinic |  |
| Kansai Medical University Hospital | Kansai Medical University Hospital Institutional Review Board |
| Shimizu Clinic | Non-Profit Organization Tokyo Allergy and Respiratory Disease Research Institute Institutional Review Board |
| Kawamura Mental Clinic | Medical Corporation Shintokai Yokohama Minoru Clinic Institutional Review Board |
| Mental Forest Clinic |  |
| Sapporo Kotoni Mental Clinic |  |
| Hokudai-dori Mental Health Clinic LiveForest Healthcare Cooperation |  |
| Kuromatsu Medical Clinic SUGIMOTO |  |
| Mental Clinic Minami |  |
| Abe Clinic |  |
| Tatsuta Clinic |  |
| Kaku Mental Clinic |  |
| Hiro Mental Clinic |  |
| Hirota Clinic |  |
| Kokura Mental Clinic |  |
| BESLI CLINIC | YOGA ALLERGY CLINIC Institutional Review Board |
| Hotei Hospital | Nakameguro Atlas Clinic Institutional Review Board |
| HIKARI CLINIC |  |
| Ujina Mental Clinic |  |
| Mukainada Ekimae Mental Clinic |  |
| Cerisier Heart Clinic |  |
| Yoyoginomori Mental Clinic | Yoyogi Mental Clinic Institutional Review Board |
| Sakurazaka Clinic SophyAnce |  |
| Jimbocho Mental Clinic |  |
| Kitaikebukuro Kokoro No Clinic |  |
| Harai Clinic |  |
| Yoyogi Mental Clinic |  |
| Murakami Hospital |  |
| Yutaka Clinic |  |
| Yokohama Onoecho Clinic |  |
| Musashikosugi Cocoromi Clinic |  |
| Mental Clinic Sakurazaka | Mental Clinic Sakurazaka Institutional Review Board |
| Jinbo Kokorono Clinic |  |
| Kokoro No Clinic Hirao |  |
| NISHIE CLINIC |  |
| Aisakura Clinic |  |
| FOREST GARDEN CLINIC |  |
| AK Clinic |  |
| COCOKARA CLINIC |  |
| Rainbow & Sea Hospital |  |
| Yuge Hospital | IRB of Yuge Hospital |
| Yamaguchi University Hospital | Yamaguchi University Hospital Institutional Review Board |
| Etoh Mental Clinic Meguro | Tokyo-Eki Center-building Clinic Institutional Review Board |
| HIGASHI-SAPPORO MENTAL CLINIC | Sapporo Psychotropic Drug Institutional Review Board |
| Ishikawa Mental Clinic |  |
| Shimode Mental Clinic | Review Board of Human Rights and Ethics for Clinical Studies Institutional Review Board |
| Meguro Station East Exit Mental Clinic |  |
| Someikai Kanagami Clinic |  |
| Hokkaido University Hospital | Hokkaido University Hospital Institutional Review Board |
| Kansai Medical University Medical Center | Kansai Medical University Medical Center  Institutional Review Board |

**Table S2:** Discontinuation of study drug and patient discontinuation/withdrawal criteria.

| 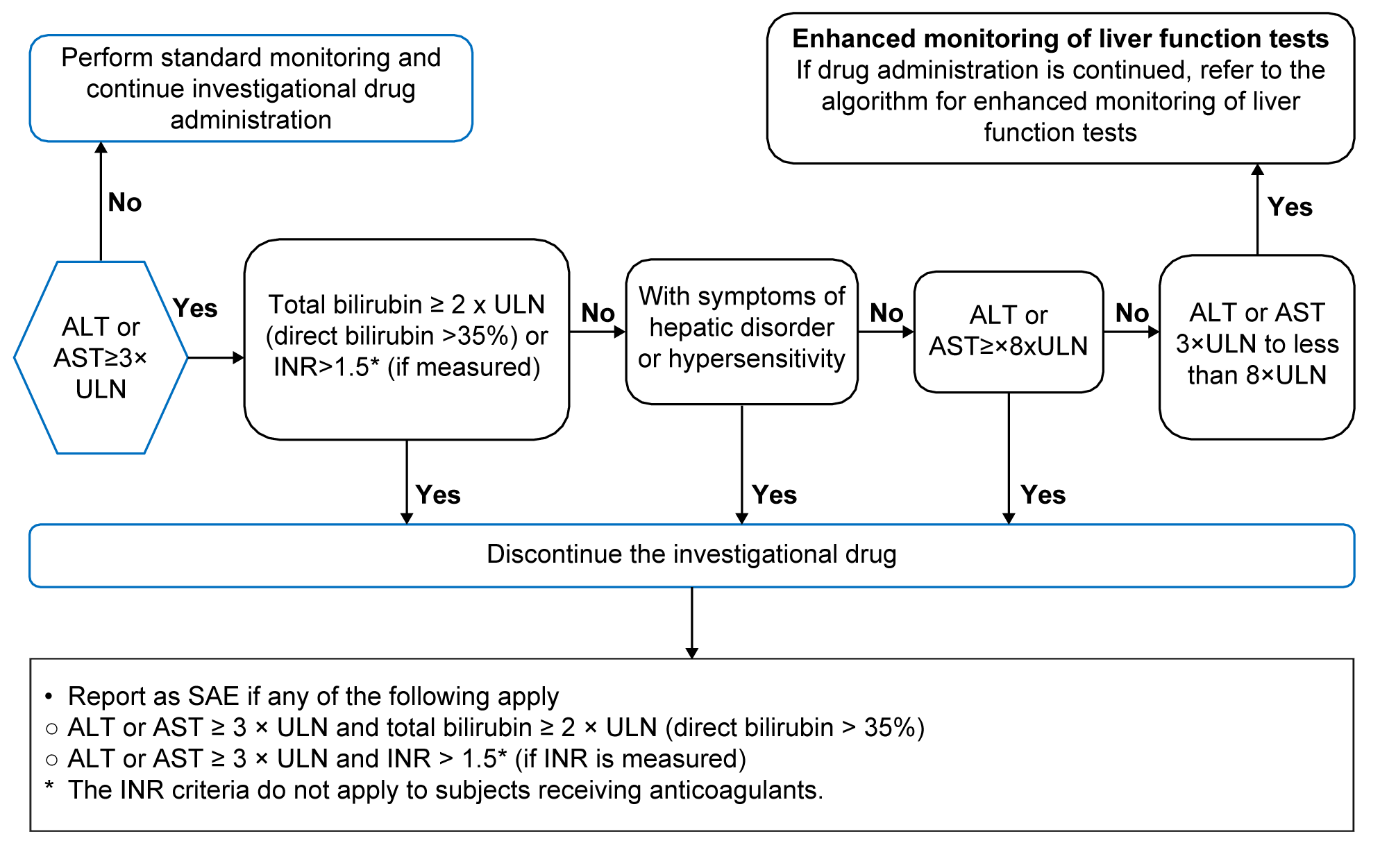  **QTc stopping criteria**   - Discontinuation criteria for QTc   - If a 12-lead ECG test confirms a clinically significant finding after patient enrollment in Part A, the investigator or qualified person will determine whether the patient is able to continue the study and whether a change in patient control is required. Clinically relevant observations include, but are not limited to, change from baseline in QT QTcF. This 12-lead ECG reading result should be recorded. All new clinically significant observations are reported as AEs.   - If any of the following conditions is met for a mean of triplicate 12-lead ECG tests, administration of the investigational drug will be discontinued:   - QTcF > 500 ms   - Change from baseline in QTcF > 60 ms - For the patients with bundle branch block, the following criteria for discontinuation shall be followed:  \| **Baseline QTc for bundle branch block** \| **Discontinuation criteria for QTc in bundle branch block** \| \| --- \| --- \| \| **< 450 ms** \| **> 500 ms** \| \| **450–480 ms** \| **≥ 530 ms** \|   **Discontinuation of study intervention due to pregnancy**   - The investigator or subinvestigator should collect pregnancy data on female patients and male patients on female partners (if applicable) during the course of administration of the investigational product and at least 26 days after the last dose of the investigational product. When a pregnancy is reported, the investigator or subinvestigator will record pregnancy data on the case follow-up form and submit it to the sponsor within 24 h after informing them of the female patient or the male/female partner (after obtaining the required informed consent from the female partner). - Pregnancy is not considered to be an AE or a serious AE but pregnancy is reported as an AE or serious morbidity or elective termination for medical reasons. - If pregnancy and outcome are abnormal (e.g., abortion spontaneous, fetal death, stillbirth, congenital anomaly, ectopic pregnancy), it is considered a serious AE and reported as such. - Patients/pregnant female partners will be followed up to confirm the outcome of the pregnancy. The principal investigator (sub-investigator) will collect follow-up information on patients/pregnant female partners and newborns and report it to the sponsor - If the investigator or subinvestigator knows that a serious AE related to pregnancy has occurred after completion of the study and considers that there is a reasonable possibility of a causal relationship with the investigational product, he/she will report it to the sponsor. Although the investigator (or subinvestigator) is not obliged to actively collect such information as in patients/pregnancy-partnered women after completion of the study, he/she may know of a serious AE from voluntary reports. - Women who are known to be pregnant while participating in the study will discontinue treatment or discontinue the study.   **Discontinuation of study intervention for other reasons**  Discontinuation of other investigational products   - The investigator or subinvestigator will discontinue study treatment in a patient and the patient should discontinue the study if any of the following are met: - When a serious or intolerable adverse event occurs and the investigator or subinvestigator judges the patient to be withdrawn from the study - In the event of a request by the patient to withdraw - When the patient is found to be inappropriate for this clinical trial after commencement of administration of the investigational drug - When patient follow-up is no longer available - In the event of the patient’s death - When the investigator determines that treatment other than that specified in the study protocol is necessary for the disease (depression) - When the investigator determines that the patient should be discontinued for other reasons   **Patient discontinuation/withdrawal from the study**   - Patients may discontinue participation in a clinical trial at any time of their own volition. The investigator may also terminate the patient’s participation in the study at any time for reasons of the patient’s safety, behavior, compliance with study procedures, or study management. - If the investigator determines that the patient requires treatment to improve the primary disease (e.g., initiation of administration of ADT, anxiolytics, sleeping pills, etc., or addition of ongoing concomitant therapy), the investigator will decide to terminate the patient’s participation and have the patient stop taking the study drug before starting the patient’s treatment. If the patient withdraws his/her consent, the investigator will not be able to administer the investigational drug. Even if the patient withdraws consent, the sponsor may retain and continue to use the data collected prior to the withdrawal of consent. - A patient who discontinues participation in a clinical trial may request that any specimens collected but not yet tested be destroyed. The investigator must record this in the clinical trial record at the site. Upon discontinuation of participation in a clinical trial (except for discontinuation at the sponsor's discretion), patients must visit the site for a discontinuation visit within 3 days if possible. All patients who discontinue due to AEs will be followed until the AEs have resolved, until the investigator determines that the patient’s symptoms have stabilized, until the patient can no longer be contacted, or until it can be determined that follow-up is no longer necessary. - The date of completion or discontinuation and the reason for discontinuation will be recorded in the eCRF. Data to be collected and assessments to be performed at the time of discontinuation of participation in the clinical trial are described in the Clinical Trial Schedule for the actions to be taken upon discontinuation of participation at the sponsor’s discretion when the number of patients who will complete the evaluation for at least 1 year (52 weeks) after the first dose of Part B is 100 or more.   **Lost to follow-up**   - A patient will be considered lost to follow-up if he/she does not present at the specified visit and the investigator or study collaborator is unable to contact them. - If a patient does not present at the specified visit, the following measures must be taken: - The investigator, subinvestigator, or study collaborator will inform the patient as soon as possible, reschedule the missed visits, explain to the patient the importance of adhering to the ordered visit schedule, and confirm the patient’s willingness and/or appropriateness to continue the study. - Prior to determining that a patient is lost to follow-up, the investigator or designee will try to contact the patient again by telephone, and the measures taken to contact the patient will be documented in the patient medical records. - A patient is considered to have discontinued participation in the study if he/she cannot be contacted continuously.   **Documentation of prior therapy/concomitant therapy**   - The investigator or subinvestigator will record all drugs and vaccines (e.g., OTC drugs, prescription drugs, vitamins, herbal supplements) used by patients during the study (Day 1 or later) in eCRF as well as information listed below. For the drugs used prior to Day 1 of Part A, all drugs used after the onset of the current episode to treat the disease (depression) shall be investigated. - Reason for use - Date of administration, including the starting date and completion date of administration - Information on mode of administration, including route of administration   **Prohibition of prior therapy/concomitant therapy**   - [Part A] - The use of the following drugs is prohibited from 28 days prior to Day 1 to the completion or discontinuation of Part A. This drug (zuranolone) is metabolized by cytochrome P450 (CYP) 3A, which may lead to increased/decreased blood drug levels:   - CYP3A strong inhibitor   - CYP3A strong inducer - The following drugs and therapies are prohibited from 14 days prior to Day 1 to the completion or discontinuation of Part A:   - ADT   - Anxiolytics   - Hypnotics (excluding non-GABA hypnotics)   - Antipsychotics   - Anti-Parkinson drugs   - Antiepileptics   - Mood stabilizers (lithium carbonate, carbamazepine, sodium valproate, lamotrigine, etc.)   - Other GABA_A_ receptor modulators or drugs that affect GABA receptor and GABA levels   - Drugs, Chinese herbal medicines, and supplements indicated for depression, depressive state (including synonyms such as depressed mood), or anxiety   - Device-based therapies such as electroconvulsive therapy and transcranial magnetic therapy   - Opioid preparations   **Restriction of pretreatment/combination treatment**   - [Part A] - The following drugs can be administered for at least twice a week from the time of informed consent to completion or discontinuation of Part A. However, the use is prohibited on the day before the individual visit:   - Non-GABA hypnotics (ramelteon, suvorexant, lemborexant, etc.)   - Drugs and Chinese herbal medicines used for insomnia treatment not meeting the above prohibited concomitant medication (antihistamines, etc.) - Addition of new therapies intended to treat depression is prohibited from informed consent to the completion or discontinuation of Part A. Therapies that are continued 14 days or more prior to Day 1 can be continued but should be applied without changing the dosage and administration until the completion of the study.   **Risk assessment of drug dependence and drug abuse**   - The Drug Dependence Assessment Committee assessed all cases of possible drug dependence using the Dependence-2A (D-2-A; visits 2, 3, and 4 of treatment period and termination visit of the treatment period) or Dependence-2B (D-2-B; termination visit of the follow-up period) scores (very much, quite a bit, a little bit, and not at all). Data for participants who scored “very much” or “quite a bit” on certain questions of the D-2-A or D-2-B assessments were submitted to the Committee. The Committee reviewed these data along with participants’ conditions; Drug Effect Questionnaire-5 (DEQ-5) data; efficacy data, including HAMD-17 and PHQ-9 total scores; and other safety data - Any response other than “not at all” had to be assessed as to whether it was attributable to the efficacy of the study intervention. - The results and date of assessment had to be documented in the eCRF. Any abnormalities detected during the assessment had to be recorded as AEs in the eCRF. |
| --- | --- | --- | --- | --- | --- | --- |
| PK evaluations:  Approximately 3 mL of plasma samples were collected for the determination of plasma levels of zuranolone. Instructions for collection and handling of plasma samples were provided separately by the sponsor in the procedural manual. The dosing time, record on the confirmation of the dosing time (within 1 h after dinner, within 1 h after snacking, or other), the date and time of sample collection, and the time of last meal before the last blood sampling were recorded in the eCRF. The date and time were recorded in a 24-h format.  The plasma samples were used for pharmacokinetic (PK) analysis of zuranolone. Each plasma sample was divided into 2 aliquots (1 each for PK analysis and backup). The samples to assess plasma levels of zuranolone could be used for the safety or efficacy assessment during or after the study in case required. Plasma drug levels were reported from the Contact Research Organization for PK analysis to unblinded staff of the sponsor after the completion of the follow-up period in Part A (Day 57 onwards). Data on the drug levels were kept confidential to the site or blinded personnel until the study was unblinded. For the PK analysis set, plasma zuranolone levels, together with the time from administration of zuranolone just before blood collection to the exact time of blood collection, were summarized in a tabular format for each treatment group and each analysis time point. In addition, the time from administration of zuranolone just before blood collection to the exact time of blood collection and plasma zuranolone levels were visualized as a graph. Furthermore, plasma zuranolone levels were summarized for each of the analysis time points. As summary statistic, n, arithmetic mean (mean), SD and its CV% (calculated as CV% = SD/mean × 100), geometric mean and its CV% (calculated as CV% geometric mean = [exp (sd2) − 1]1/2 × 100, wherein "sd" refers to standard deviation of the value converted to natural log value), median, min, and max were calculated. When handling the plasma zuranolone levels BLQ, BLQ was regarded as 0 to calculate the mean, SD, CV%, min, median, and max. BLQ was excluded for the calculation of the geometric mean and CV% geometric mean.  After the plasma levels were measured, data that could be clearly explained as inadequate for the analysis by the sponsor's PK analysis personnel were excluded. In case of exclusion, the reason for exclusion was described in the clinical study report. Population PK analyses were performed, as and when feasible, on a nonlinear mixed-effect model using NONMEM Version 7.3 or higher. Population PK analyses were performed based on the protocol that was prepared separately and reported in a separate population PK analysis report. |

ADT, antidepressant; AE, adverse event; ALT, alanine aminotransferase; AST, aspartate aminotransferase; BLQ, below the lower limit of quantitation; CV%, coefficient of variation; CYP, cytochrome P450; ECG, electrocardiogram; eCRF, electronic case report form; GABA, gamma-aminobutyric acid; INR, international normalized ratio; OTC, over-the-counter; PK, pharmacokinetic; QTc, corrected QT interval; QTcF, corrected QT interval using Fridericia’s formula; SD, standard deviation; ULN, upper limit of normal.

**Table S3:** Analysis of CGI-S by timepoint (full analysis set)

| Time point | Treatment group | n^a^ | “Normal, not at all ill” or “borderline mentally ill” of CGI-S^b^ n (%) | Improvement rate (%) | Comparison with placebo | |
| --- | --- | --- | --- | --- | --- | --- |
|  |  |  |  |  | Adjusted OR | P-value |
|  |  |  |  |  | [95% CI] |  |
| Day 3 | Zuranolone 30 mg | 201 | 13 | 6.5 | 4.55 [1.28, 16.16] | 0.0193 |
|  | Placebo | 199 | 3 | 1.5 | --- | --- |
| Day 8 | Zuranolone 30 mg | 198 | 39 | 19.7 | 2.74 [1.47, 5.09] | 0.0015 |
|  | Placebo | 196 | 16 | 8.2 | --- | --- |
| Day 15 | Zuranolone 30 mg | 192 | 60 | 31.3 | 1.29 [0.83, 2.02] | 0.253 |
|  | Placebo | 194 | 51 | 26.3 | --- | --- |
| Day 22 | Zuranolone 30 mg | 187 | 60 | 32.1 | 1.18 [0.76, 1.82] | 0.4644 |
|  | Placebo | 195 | 56 | 28.7 | --- | --- |
| Day 29 | Zuranolone 30 mg | 185 | 65 | 35.1 | 1.33 [0.85, 2.06] | 0.2113 |
|  | Placebo | 192 | 56 | 29.2 | --- | --- |
| Day 36 | Zuranolone 30 mg | 179 | 60 | 33.5 | 0.95 [0.61, 1.47] | 0.8183 |
|  | Placebo | 189 | 65 | 34.4 | --- | --- |
| Day 43 | Zuranolone 30 mg | 178 | 68 | 38.2 | 1.23 [0.79, 1.91] | 0.3543 |
|  | Placebo | 188 | 63 | 33.5 | --- | --- |
| Day 50 | Zuranolone 30 mg | 171 | 62 | 36.3 | 1.15 [0.74, 1.80] | 0.5271 |
|  | Placebo | 187 | 62 | 33.2 | --- | --- |
| Day 57 | Zuranolone 30 mg | 175 | 61 | 34.9 | 1.33 [0.84, 2.10] | 0.2224 |
|  | Placebo | 184 | 54 | 29.3 | --- | --- |

^a^Number of patients with nonmissing CGI-S score at the visit.

The denominator of the percentage is the number of patients with nonmissing CGI-S score at the visit.

^b^The criteria for CGI-S were categorized as normal, not at all ill, borderline mentally ill, mildly ill, moderately ill, markedly ill, severely ill, and among the most extremely ill. Of these, rating of “normal, not at all ill” or “borderline mentally ill” was defined as “normal/borderline mentally ill”

IPW-GEE with an independent structure for the working correlation matrix was applied to estimate study intervention difference between the zuranolone 30 mg and placebo groups; Fixed effects: treatment group, time point, and interaction effect (treatment group and time point). Covariates: baseline value of the HAMD-17 total score, sex, and presence or absence of prior drug for depressive episodes.

Abbreviations: CGI-S, Clinical Global Impression–Severity of Illness; CI, confidence interval; HAMD-17, 17-item Hamilton Rating Scale for Depression; IPW-GEE, inverse probability-weighted generalized estimating equation; OR, odds ratio.

**Table S4.** Changes from baseline in the ISI total score by timepoint (full analysis set)

| Time point | Treatment group | n | Mean  (SD) | Least-squares mean change | Comparison with placebo | |
| --- | --- | --- | --- | --- | --- | --- |
|  |  |  |  | from baseline (SE) | Difference in adjusted mean | P-value |
|  |  |  |  |  | [95% CI] |  |
| Baseline | Zuranolone 30 mg | 204 | 17.2 (4.9) | --- | --- | --- |
|  | Placebo | 199 | 17.5 (5.3) | --- | --- | --- |
| Day 3 | Zuranolone 30 mg | 204 | 16.5 (5.3) | −0.80 (0.20) | −0.66 [−1.22, −0.10] | 0.0217 |
|  | Placebo | 199 | 17.4 (5.5) | −0.15 (0.20) | --- | --- |
| Day 8 | Zuranolone 30 mg | 202 | 14.4 (5.6) | −2.86 (0.29) | −1.32 [−2.12, −0.52] | 0.0013 |
|  | Placebo | 198 | 16.0 (5.7) | −1.54 (0.29) | --- | --- |
| Day 15 | Zuranolone 30 mg | 198 | 13.0 (6.0) | −4.17 (0.34) | −1.41 [−2.36, −0.45] | 0.0039 |
|  | Placebo | 198 | 14.8 (6.2) | −2.76 (0.34) | --- | --- |
| Day 22 | Zuranolone 30 mg | 191 | 13.6 (6.1) | −3.49 (0.35) | −0.31 [−1.27, 0.65] | 0.5212 |
|  | Placebo | 197 | 14.3 (6.4) | −3.18 (0.34) | --- | --- |
| Day 29 | Zuranolone 30 mg | 187 | 13.3 (6.7) | −3.74 (0.38) | −0.67 [−1.73, 0.39] | 0.2129 |
|  | Placebo | 195 | 14.4 (6.5) | −3.07 (0.38) | --- | --- |
| Day 36 | Zuranolone 30 mg | 182 | 13.3 (6.9) | −3.78 (0.39) | −0.73 [−1.81, 0.35] | 0.1846 |
|  | Placebo | 192 | 14.5 (6.7) | −3.05 (0.39) | --- | --- |
| Day 43 | Zuranolone 30 mg | 182 | 13.1 (6.6) | −4.01 (0.39) | −0.67 [−1.75, 0.42] | 0.2265 |
|  | Placebo | 192 | 14.2 (6.8) | −3.35 (0.39) | --- | --- |
| Day 50 | Zuranolone 30 mg | 182 | 13.0 (7.1) | −4.26 (0.40) | −1.36 [−2.47, −0.25] | 0.0169 |
|  | Placebo | 189 | 14.6 (6.8) | −2.90 (0.40) | --- | --- |
| Day 57 | Zuranolone 30 mg | 180 | 13.1 (7.3) | −4.12 (0.42) | −0.91 [−2.07, 0.25] | 0.123 |
|  | Placebo | 190 | 14.3 (6.7) | −3.20 (0.41) | --- | --- |
|  |  |  |  |  |  |  |

MMRM with an unstructured covariance structure was applied to estimate study intervention difference between the zuranolone 30 mg and placebo groups; Fixed effects: treatment group, time point, and interaction effect (treatment group and time point). Covariates: baseline value of the HAMD-17 total score, sex, and presence or absence of prior drug for depressive episodes.

Abbreviations: CI, confidence interval; HAMD-17, 17-item Hamilton Rating Scale for Depression; ISI, insomnia severity index; MMRM, mixed-effects model for repeated measures; SD, standard deviation: SE, standard error.

**Table S5.** TEAEs during the treatment and follow-up periods (safety analysis set)

| System Organ Class^†^ - Preferred Term | **Zuranolone 30 mg** | | | | **Placebo** | | | |
| --- | --- | --- | --- | --- | --- | --- | --- | --- |
|  | **Treatment period *N* = 205** | | **Follow-up period *N* = 205** | | **Treatment period *N* = 199** | | **Follow-up**  **period *N* = 199** | |
|  | ***n* (%)** | **Event** | ***n* (%)** | **Event** | ***n* (%)** | **Event** | ***n* (%)** | **Event** |
| Participants with any TEAE | 96 (46.8) | 144 | 50 (24.4) | 73 | 45 (22.6) | 57 | 48 (24.1) | 64 |
| Infections and infestations | 12 (5.9) | 12 | 14 (6.8) | 15 | 8 (4.0) | 9 | 14 (7.0) | 14 |
| - Upper pharyngitis | 2 (1.0) | 2 | 6 (2.9) | 6 | 3 (1.5) | 3 | 3 (1.5) | 3 |
| - COVID-19 | 5 (2.4) | 5 | 3 (1.5) | 3 | 3 (1.5) | 3 | 6 (3.0) | 6 |
| - Gastroenteritis | 0 | 0 | 2 (1.0) | 2 | 0 | 0 | 0 | 0 |
| - Urinary tract infection | 2 (1.0) | 2 | 0 | 0 | 3 (1.5) | 3 | 1 (0.5) | 1 |
| - Herpes simplex | 1 (0.5) | 1 | 0 | 0 | 0 | 0 | 0 | 0 |
| - Herpes zoster | 1 (0.5) | 1 | 0 | 0 | 0 | 0 | 0 | 0 |
| - Hordeolum | 0 | 0 | 1 (0.5) | 1 | 0 | 0 | 0 | 0 |
| - Mumps | 0 | 0 | 1 (0.5) | 1 | 0 | 0 | 0 | 0 |
| - Otitis media | 0 | 0 | 1 (0.5) | 1 | 0 | 0 | 0 | 0 |
| - Subcutaneous abscess | 1 (0.5) | 1 | 0 | 0 | 0 | 0 | 0 | 0 |
| - Candida infection | 0 | 0 | 1 (0.5) | 1 | 0 | 0 | 0 | 0 |
| - Bacteriuria | 0 | 0 | 0 | 0 | 0 | 0 | 1 (0.5) | 1 |
| - Herpes virus infection | 0 | 0 | 0 | 0 | 0 | 0 | 1 (0.5) | 1 |
| - Influenza | 0 | 0 | 0 | 0 | 0 | 0 | 1 (0.5) | 1 |
| - Pharyngitis | 0 | 0 | 0 | 0 | 0 | 0 | 1 (0.5) | 1 |
| Blood and lymphatic system disorders | 0 | 0 | 1 (0.5) | 1 | 0 | 0 | 0 | 0 |
| - Leukocytosis | 0 | 0 | 1 (0.5) | 1 | 0 | 0 | 0 | 0 |
| Immune system disorders | 0 | 0 | 1 (0.5) | 1 | 0 | 0 | 0 | 0 |
| - Arthropod sting allergic | 0 | 0 | 1 (0.5) | 1 | 0 | 0 | 0 | 0 |
| Metabolism and nutrition disorders | 2 (1.0) | 2 | 1 (0.5) | 1 | 1 (0.5) | 1 | 2 (1.0) | 2 |
| - Decreased appetite | 2 (1.0) | 2 | 0 | 0 | 0 | 0 | 0 | 0 |
| - Hypertriglyceridaemia | 0 | 0 | 1 (0.5) | 1 | 0 | 0 | 0 | 0 |
| - Gout | 0 | 0 | 0 | 0 | 0 | 0 | 1 (0.5) | 1 |
| - Hyperuricaemia | 0 | 0 | 0 | 0 | 0 | 0 | 1 (0.5) | 1 |
| - Increased appetite | 0 | 0 | 0 | 0 | 1 (0.5) | 1 | 0 | 0 |
| Psychiatric disorders | 5 (2.4) | 5 | 0 | 0 | 1 (0.5) | 1 | 1 (0.5) | 1 |
| - Insomnia | 2 (1.0) | 2 | 0 | 0 | 1 (0.5) | 1 | 1 (0.5) | 1 |
| - Nightmares | 2 (1.0) | 2 | 0 | 0 | 0 | 0 | 0 | 0 |
| - Agitation | 1 (0.5) | 1 | 0 | 0 | 0 | 0 | 0 | 0 |
| Nervous system disorders | 52 (25.4) | 64 | 6 (2.9) | 6 | 15 (7.5) | 17 | 3 (1.5) | 3 |
| - Somnolence | 27 (13.2) | 27 | 0 | 0 | 11 (5.5) | 11 | 1 (0.5) | 1 |
| - Dizziness | 25 (12.2) | 25 | 1 (0.5) | 1 | 2 (1.0) | 2 | 1 (0.5) | 1 |
| - Headache | 7 (3.4) | 7 | 3 (1.5) | 3 | 3 (1.5) | 3 | 1 (0.5) | 1 |
| - Disturbance in attention | 1 (0.5) | 1 | 0 | 0 | 0 | 0 | 0 | 0 |
| - Dysarthria | 1 (0.5) | 1 | 0 | 0 | 0 | 0 | 0 | 0 |
| - Hypoaesthesia | 0 | 0 | 1 (0.5) | 1 | 0 | 0 | 0 | 0 |
| - Sensory disturbance | 1 (0.5) | 1 | 0 | 0 | 0 | 0 | 0 | 0 |
| - Tremor | 1 (0.5) | 1 | 0 | 0 | 0 | 0 | 0 | 0 |
| - Intercostal neuralgia | 0 | 0 | 1 (0.5) | 1 | 0 | 0 | 0 | 0 |
| - Restless legs syndrome | 1 (0.5) | 1 | 0 | 0 | 0 | 0 | 0 | 0 |
| - Head discomfort | 0 | 0 | 0 | 0 | 1 (0.5) | 1 | 0 | 0 |
| Eye disorders | 0 | 0 | 0 | 0 | 1 (0.5) | 1 | 2 (1.0) | 2 |
| - Cataract | 0 | 0 | 0 | 0 | 0 | 0 | 1 (0.5) | 1 |
| - Conjunctivitis allergic | 0 | 0 | 0 | 0 | 1 (0.5) | 1 | 0 | 0 |
| - Vitreous floaters | 0 | 0 | 0 | 0 | 0 | 0 | 1 (0.5) | 1 |
| Ear and labyrinth disorders | 4 (2.0) | 4 | 0 | 0 | 0 | 0 | 1 (0.5) | 1 |
| - Vertigo | 4 (2.0) | 4 | 0 | 0 | 0 | 0 | 0 | 0 |
| - Tinnitus | 0 | 0 | 0 | 0 | 0 | 0 | 1 (0.5) | 1 |
| Cardiac disorders | 1 (0.5) | 1 | 1 (0.5) | 1 | 0 | 0 | 3 (1.5) | 3 |
| - Palpitations | 1 (0.5) | 1 | 0 | 0 | 0 | 0 | 0 | 0 |
| - Sinus bradycardia | 0 | 0 | 1 (0.5) | 1 | 0 | 0 | 0 | 0 |
| - Bradycardia | 0 | 0 | 0 | 0 | 0 | 0 | 1 (0.5) | 1 |
| - Supraventricular extrasystoles | 0 | 0 | 0 | 0 | 0 | 0 | 1 (0.5) | 1 |
| - Ventricular extrasystoles | 0 | 0 | 0 | 0 | 0 | 0 | 1 (0.5) | 1 |
| Vascular disorders | 1 (0.5) | 1 | 1 (0.5) | 1 | 0 | 0 | 1 (0.5) | 1 |
| - Orthostatic hypotension | 1 (0.5) | 1 | 1 (0.5) | 1 | 0 | 0 | 0 | 0 |
| - Hypertension | 0 | 0 | 0 | 0 | 0 | 0 | 1 (0.5) | 1 |
| Respiratory, thoracic and mediastinal disorders | 0 | 0 | 1 (0.5) | 1 | 0 | 0 | 2 (1.0) | 2 |
| - Oropharyngeal pain | 0 | 0 | 1 (0.5) | 1 | 0 | 0 | 0 | 0 |
| - Cough | 0 | 0 | 0 | 0 | 0 | 0 | 2 (1.0) | 2 |
| Gastrointestinal disorders | 16 (7.8) | 16 | 11 (5.4) | 17 | 12 (6.0) | 14 | 5 (2.5) | 8 |
| - Diarrhoea | 3 (1.5) | 3 | 5 (2.4) | 5 | 3 (1.5) | 3 | 1 (0.5) | 1 |
| - Nausea | 7 (3.4) | 7 | 1 (0.5) | 2 | 2 (1.0) | 2 | 0 | 0 |
| - Vomiting | 2 (1.0) | 2 | 2 (1.0) | 2 | 0 | 0 | 1 (0.5) | 1 |
| - Abdominal pain upper | 1 (0.5) | 1 | 1 (0.5) | 1 | 2 (1.0) | 2 | 1 (0.5) | 1 |
| - Constipation | 0 | 0 | 2 (1.0) | 3 | 3 (1.5) | 3 | 2 (1.0) | 2 |
| - Abdominal discomfort | 1 (0.5) | 1 | 0 | 0 | 1 (0.5) | 1 | 1 (0.5) | 1 |
| - Abdominal pain | 0 | 0 | 1 (0.5) | 1 | 0 | 0 | 0 | 0 |
| - Colitis ischaemic | 0 | 0 | 1 (0.5) | 1 | 0 | 0 | 0 | 0 |
| - Dental caries | 1 (0.5) | 1 | 0 | 0 | 0 | 0 | 0 | 0 |
| - Haemorrhoids | 0 | 0 | 1 (0.5) | 1 | 1 (0.5) | 1 | 0 | 0 |
| - Retching | 0 | 0 | 1 (0.5) | 1 | 0 | 0 | 0 | 0 |
| - Gastrointestinal hypermotility | 1 (0.5) | 1 | 0 | 0 | 0 | 0 | 0 | 0 |
| - Abdominal distension | 0 | 0 | 0 | 0 | 1 (0.5) | 1 | 0 | 0 |
| - Periodontal disease | 0 | 0 | 0 | 0 | 0 | 0 | 1 (0.5) | 1 |
| - Anal haemorrhage | 0 | 0 | 0 | 0 | 1 (0.5) | 1 | 0 | 0 |
| - Large intestine polyp | 0 | 0 | 0 | 0 | 0 | 0 | 1 (0.5) | 1 |
| Hepatobiliary disorders | 0 | 0 | 1 (0.5) | 1 | 0 | 0 | 0 | 0 |
| - Hepatic steatosis | 0 | 0 | 1 (0.5) | 1 | 0 | 0 | 0 | 0 |
| Skin and subcutaneous tissue disorders | 0 | 0 | 3 (1.5) | 3 | 1 (0.5) | 1 | 5 (2.5) | 5 |
| - Eczema | 0 | 0 | 1 (0.5) | 1 | 0 | 0 | 1 (0.5) | 1 |
| - Pruritus | 0 | 0 | 1 (0.5) | 1 | 0 | 0 | 2 (1.0) | 2 |
| - Rash | 0 | 0 | 1 (0.5) | 1 | 1 (0.5) | 1 | 0 | 0 |
| - Dry skin | 0 | 0 | 0 | 0 | 0 | 0 | 1 (0.5) | 1 |
| - Urticaria | 0 | 0 | 0 | 0 | 0 | 0 | 1 (0.5) | 1 |
| Musculoskeletal and connective tissue disorders | 3 (1.5) | 3 | 6 (2.9) | 6 | 1 (0.5) | 1 | 4 (2.0) | 4 |
| - Myalgia | 1 (0.5) | 1 | 4 (2.0) | 4 | 1 (0.5) | 1 | 1 (0.5) | 1 |
| - Back pain | 1 (0.5) | 1 | 1 (0.5) | 1 | 0 | 0 | 1 (0.5) | 1 |
| - Muscular weakness | 1 (0.5) | 1 | 0 | 0 | 0 | 0 | 0 | 0 |
| - Musculoskeletal pain | 0 | 0 | 1 (0.5) | 1 | 0 | 0 | 0 | 0 |
| - Temporomandibular joint syndrome | 0 | 0 | 0 | 0 | 0 | 0 | 1 (0.5) | 1 |
| - Fibromyalgia | 0 | 0 | 0 | 0 | 0 | 0 | 1 (0.5) | 1 |
| Renal and urinary disorders | 0 | 0 | 0 | 0 | 1 (0.5) | 1 | 0 | 0 |
| - Pollakiuria | 0 | 0 | 0 | 0 | 1 (0.5) | 1 | 0 | 0 |
| Reproductive system and breast disorders | 1 (0.5) | 1 | 1 (0.5) | 1 | 0 | 0 | 0 | 0 |
| - Dysmenorrhoea | 1 (0.5) | 1 | 1 (0.5) | 1 | 0 | 0 | 0 | 0 |
| General disorders and administration site conditions | 25 (12.2) | 28 | 10 (4.9) | 11 | 4 (2.0) | 4 | 5 (2.5) | 7 |
| - Feeling abnormal | 13 (6.3) | 15 | 0 | 0 | 1 (0.5) | 1 | 0 | 0 |
| - Pyrexia | 3 (1.5) | 3 | 7 (3.4) | 8 | 0 | 0 | 3 (1.5) | 3 |
| - Asthenia | 4 (2.0) | 4 | 1 (0.5) | 1 | 0 | 0 | 0 | 0 |
| - Malaise | 2 (1.0) | 2 | 1 (0.5) | 1 | 2 (1.0) | 2 | 1 (0.5) | 1 |
| - Chest pain | 1 (0.5) | 1 | 0 | 0 | 0 | 0 | 1 (0.5) | 1 |
| - Feeling drunk | 1 (0.5) | 1 | 0 | 0 | 0 | 0 | 0 | 0 |
| - Gait disturbance | 1 (0.5) | 1 | 0 | 0 | 0 | 0 | 0 | 0 |
| - Injection site bruising | 0 | 0 | 1 (0.5) | 1 | 1 (0.5) | 1 | 0 | 0 |
| - Foot tangles | 1 (0.5) | 1 | 0 | 0 | 0 | 0 | 0 | 0 |
| - Injection site pain | 0 | 0 | 0 | 0 | 0 | 0 | 1 (0.5) | 1 |
| - Pain | 0 | 0 | 0 | 0 | 0 | 0 | 1 (0.5) | 1 |
| Investigations | 3 (1.5) | 4 | 5 (2.4) | 5 | 7 (3.5) | 7 | 6 (3.0) | 7 |
| - Blood pressure decreased | 1 (0.5) | 1 | 1 (0.5) | 1 | 0 | 0 | 0 | 0 |
| - Alanine aminotransferase increased | 1 (0.5) | 1 | 0 | 0 | 1 (0.5) | 1 | 2 (1.0) | 2 |
| - Aspartate aminotransferase increased | 0 | 0 | 1 (0.5) | 1 | 0 | 0 | 1 (0.5) | 1 |
| - Blood cholesterol increased | 0 | 0 | 1 (0.5) | 1 | 0 | 0 | 0 | 0 |
| - Blood creatine phosphokinase increased | 0 | 0 | 1 (0.5) | 1 | 1 (0.5) | 1 | 0 | 0 |
| - Systolic blood pressure decreased | 0 | 0 | 1 (0.5) | 1 | 0 | 0 | 0 | 0 |
| - Gamma-glutamyltransferase increased | 1 (0.5) | 1 | 0 | 0 | 0 | 0 | 0 | 0 |
| - Urobilinogen urine increased | 1 (0.5) | 1 | 0 | 0 | 0 | 0 | 0 | 0 |
| - Blood glucose increased | 0 | 0 | 0 | 0 | 1 (0.5) | 1 | 0 | 0 |
| - Blood uric acid increased | 0 | 0 | 0 | 0 | 0 | 0 | 2 (1.0) | 2 |
| - Glucose urine present | 0 | 0 | 0 | 0 | 0 | 0 | 1 (0.5) | 1 |
| - Weight decreased | 0 | 0 | 0 | 0 | 1 (0.5) | 1 | 0 | 0 |
| - Leukocyte esterase urine present | 0 | 0 | 0 | 0 | 1 (0.5) | 1 | 0 | 0 |
| - Blood in urine | 0 | 0 | 0 | 0 | 2 (1.0) | 2 | 1 (0.5) | 1 |
| Injury, poisoning and procedural complications | 3 (1.5) | 3 | 2 (1.0) | 2 | 0 | 0 | 4 (2.0) | 4 |
| - Vaccination complication | 0 | 0 | 1 (0.5) | 1 | 0 | 0 | 1 (0.5) | 1 |
| - Gingival injury | 1 (0.5) | 1 | 0 | 0 | 0 | 0 | 0 | 0 |
| - Thermal burn | 1 (0.5) | 1 | 0 | 0 | 0 | 0 | 0 | 0 |
| - Procedural pain | 1 (0.5) | 1 | 0 | 0 | 0 | 0 | 0 | 0 |
| - Skin abrasion | 0 | 0 | 1 (0.5) | 1 | 0 | 0 | 0 | 0 |
| - Arthropod sting | 0 | 0 | 0 | 0 | 0 | 0 | 1 (0.5) | 1 |
| - Heat illness | 0 | 0 | 0 | 0 | 0 | 0 | 1 (0.5) | 1 |
| - Tooth dislocation | 0 | 0 | 0 | 0 | 0 | 0 | 1 (0.5) | 1 |

Event: Number of events.

Treatment period: up to 2 weeks; follow-up period: 2-8 weeks.

The definition of *N* used in the follow-up period was the same as that used in the treatment period.

AEs that occurred after discontinuation in the treatment period were counted as events in the follow-up period.

^†^System Organ Class and Preferred Term as per MedDRA version 24.1.

Abbreviations: AE, adverse event; COVID-19, coronavirus disease 2019; MedDRA, Medical Dictionary for Regulatory Activities; TEAE, treatment‑emergent adverse event.

**Table S6:** Overall summary of the incidence of TRAEs categorized by the treatment and follow-up period (safety analysis set)

| System Organ Class^a^ - Preferred Term | **Zuranolone 30 mg** | | | | **Placebo** | | | |
| --- | --- | --- | --- | --- | --- | --- | --- | --- |
|  | **Treatment period N = 205** | | **Follow-up period N = 205** | | **Treatment period N = 199** | | **Follow-up period N = 199** | |
|  | **n (%)** | **Event** | **n (%)** | **Event** | **n (%)** | **Event** | **n (%)** | **Event** |
| Participants with any  TRAE | 70 (34.1) | 105 | 3 (1.5) | 3 | 22 (11.1) | 30 | 3 (1.5) | 4 |
| Metabolism and nutrition disorders | 2 (1.0) | 2 | 0 | 0 | 1 (0.5) | 1 | 0 | 0 |
| - Decreased appetite | 2 (1.0) | 2 | 0 | 0 | 0 | 0 | 0 | 0 |
| - Increased appetite | 0 | 0 | 0 | 0 | 1 (0.5) | 1 | 0 | 0 |
| Psychiatric disorders | 2 (1.0) | 2 | 0 | 0 | 1 (0.5) | 1 | 0 | 0 |
| - Agitation | 1 (0.5) | 1 | 0 | 0 | 0 | 0 | 0 | 0 |
| - Nightmares | 1 (0.5) | 1 | 0 | 0 | 0 | 0 | 0 | 0 |
| - Insomnia | 0 | 0 | 0 | 0 | 1 (0.5) | 1 | 0 | 0 |
| Nervous system disorders | 49 (23.9) | 60 | 2 (1.0) | 2 | 14 (7.0) | 16 | 1 (0.5) | 1 |
| - Somnolence | 27 (13.2) | 27 | 0 | 0 | 11 (5.5) | 11 | 0 | 0 |
| - Dizziness | 25 (12.2) | 25 | 1 (0.5) | 1 | 1 (0.5) | 1 | 1 (0.5) | 1 |
| - Headache | 4 (2.0) | 4 | 0 | 0 | 3 (1.5) | 3 | 0 | 0 |
| - Disturbance in attention | 1 (0.5) | 1 | 0 | 0 | 0 | 0 | 0 | 0 |
| - Dysarthria | 1 (0.5) | 1 | 0 | 0 | 0 | 0 | 0 | 0 |
| - Hypoaesthesia | 0 | 0 | 1 (0.5) | 1 | 0 | 0 | 0 | 0 |
| - Tremor | 1 (0.5) | 1 | 0 | 0 | 0 | 0 | 0 | 0 |
| - Restless legs syndrome | 1 (0.5) | 1 | 0 | 0 | 0 | 0 | 0 | 0 |
| - Head discomfort | 0 | 0 | 0 | 0 | 1 (0.5) | 1 | 0 | 0 |
| Ear and labyrinth disorders | 4 (2.0) | 4 | 0 | 0 | 0 | 0 | 0 | 0 |
| - Vertigo | 4 (2.0) | 4 | 0 | 0 | 0 | 0 | 0 | 0 |
| Cardiac disorders | 1 (0.5) | 1 | 0 | 0 | 0 | 0 | 1 (0.5) | 1 |
| - Palpitations | 1 (0.5) | 1 | 0 | 0 | 0 | 0 | 0 | 0 |
| - Supraventricular extrasystoles | 0 | 0 | 0 | 0 | 0 | 0 | 1 (0.5) | 1 |
| Gastrointestinal disorders | 11 (5.4) | 11 | 0 | 0 | 7 (3.5) | 8 | 0 | 0 |
| - Nausea | 6 (2.9) | 6 | 0 | 0 | 2 (1.0) | 2 | 0 | 0 |
| - Vomiting | 2 (1.0) | 2 | 0 | 0 | 0 | 0 | 0 | 0 |
| - Abdominal discomfort | 1 (0.5) | 1 | 0 | 0 | 1 (0.5) | 1 | 0 | 0 |
| - Diarrhoea | 1 (0.5) | 1 | 0 | 0 | 2 (1.0) | 2 | 0 | 0 |
| - Gastrointestinal hypermotility | 1 (0.5) | 1 | 0 | 0 | 0 | 0 | 0 | 0 |
| - Abdominal distension | 0 | 0 | 0 | 0 | 1 (0.5) | 1 | 0 | 0 |
| - Constipation | 0 | 0 | 0 | 0 | 2 (1.0) | 2 | 0 | 0 |
| Musculoskeletal and connective tissue disorders | 1 (0.5) | 1 | 0 | 0 | 0 | 0 | 0 | 0 |
| - Muscular weakness | 1 (0.5) | 1 | 0 | 0 | 0 | 0 | 0 | 0 |
| Renal and urinary disorders | 0 | 0 | 0 | 0 | 1 (0.5) | 1 | 0 | 0 |
| - Pollakiuria | 0 | 0 | 0 | 0 | 1 (0.5) | 1 | 0 | 0 |
| General disorders and administration site conditions | 20 (9.8) | 23 | 0 | 0 | 3 (1.5) | 3 | 0 | 0 |
| - Feeling abnormal | 13 (6.3) | 15 | 0 | 0 | 1 (0.5) | 1 | 0 | 0 |
| - Asthenia | 3 (1.5) | 3 | 0 | 0 | 0 | 0 | 0 | 0 |
| - Malaise | 2 (1.0) | 2 | 0 | 0 | 2 (1.0) | 2 | 0 | 0 |
| - Feeling drunk | 1 (0.5) | 1 | 0 | 0 | 0 | 0 | 0 | 0 |
| - Gait disturbance | 1 (0.5) | 1 | 0 | 0 | 0 | 0 | 0 | 0 |
| - Foot tangles | 1 (0.5) | 1 | 0 | 0 | 0 | 0 | 0 | 0 |
| Investigations | 1 (0.5) | 1 | 1 (0.5) | 1 | 0 | 0 | 1 (0.5) | 2 |
| - Blood creatine phosphokinase increased | 0 | 0 | 1 (0.5) | 1 | 0 | 0 | 0 | 0 |
| - Blood pressure decreased | 1 (0.5) | 1 | 0 | 0 | 0 | 0 | 0 | 0 |
| - Alanine aminotransferase increased | 0 | 0 | 0 | 0 | 0 | 0 | 1 (0.5) | 1 |
| - Aspartate aminotransferase increased | 0 | 0 | 0 | 0 | 0 | 0 | 1 (0.5) | 1 |

Event: Number of events

Treatment period: Up to 2 weeks; follow-up period: 2–8 weeks

The definition of N used in the follow-up period was the same as that used in the treatment period.

AEs that occurred after discontinuation in the treatment period were counted as events in the follow-up period.

A “TRAE” was defined as an event in which causality could not be denied among AEs reported after the first administration of the study intervention.

^a^System Organ Class and Preferred Term according to MedDRA version 24.1

Abbreviations: AE, adverse event; MedDRA, Medical Dictionary for Regulatory Activities; TRAE, treatment-related adverse event

**Table S7.** Tipping point analysis for missing data of HAMD-17 total score at Day 15

| k^a^ | Vs placebo | | |
| --- | --- | --- | --- |
|  | Difference in adjusted mean (SE)^b^ | [95% CI] | P-value |
| k = 0 | –1.20 (0.57) | [–2.33, –0.07] | 0.0368 |
| k = 0.1 | –1.19 (0.57) | [–2.32, –0.07] | 0.0380 |
| k = 0.2 | –1.18 (0.57) | [–2.31, –0.06] | 0.0392 |
| k = 0.3 | –1.18 (0.57) | [–2.30, –0.05] | 0.0405 |
| k = 0.4 | –1.17 (0.57) | [–2.30, –0.04] | 0.0419 |
| k = 0.5 | –1.16 (0.57) | [–2.29, –0.04] | 0.0432 |
| k = 0.6 | –1.15 (0.57) | [–2.28, –0.03] | 0.0447 |
| k = 0.7 | –1.15 (0.57) | [–2.27, –0.02] | 0.0461 |
| k = 0.8 | –1.14 (0.57) | [–2.27, –0.01] | 0.0476 |
| k = 0.9 | –1.13 (0.57) | [–2.26, 0.00] | 0.0491 |
| k = 1 (tipping point)^c^ | –1.12 (0.58) | [–2.25, 0.00] | 0.0507 |

^a^k is the coefficient of the treatment difference between the treatment and placebo groups which was subtracted from the effect of the zuranolone 30 mg group in primary analysis with the MMRM method. All imputed data in zuranolone 30 mg group were subtracted by a delta defined as k times the treatment difference.

^b^MMRM included the data obtained until Day 15. MMRM with unstructured covariance structure was applied to estimate study intervention difference between the zuranolone 30 mg and placebo groups; change from baseline as response variable; study intervention, time point, and interaction between study intervention and time point as fixed effects; HAMD-17 total score at baseline, presence or absence of prior drug for depressive episodes and sex as covariates.

^c^The tipping point is defined as the value of k at which the upper limit of the 95% CI for the adjusted mean difference is greater than 0.

Abbreviations: CI, confidence interval; HAMD-17, 17-item Hamilton Rating Scale for Depression; MMRM, mixed-effects model for repeated measures; SE, standard error
